# A Method for Identification and Analysis of Non-Overlapping Myeloid Immunophenotypes in Humans

**DOI:** 10.1371/journal.pone.0121546

**Published:** 2015-03-23

**Authors:** Michael P. Gustafson, Yi Lin, Mary L. Maas, Virginia P. Van Keulen, Patrick B. Johnston, Tobias Peikert, Dennis A. Gastineau, Allan B. Dietz

**Affiliations:** 1 Human Cellular Therapy Laboratory, Division of Transfusion Medicine, Department of Laboratory Medicine and Pathology, Mayo Clinic, Rochester, MN, United States of America; 2 Division of Hematology, Department of Medicine, Mayo Clinic, Rochester, MN, United States of America; 3 Department of Immunology, Mayo Clinic, Rochester, MN, United States of America; 4 Pulmonary and Critical Care Medicine, Mayo Clinic, Rochester, MN, United States of America; Jackson Laboratory, Genomic Medicine, United States

## Abstract

The development of flow cytometric biomarkers in human studies and clinical trials has been slowed by inconsistent sample processing, use of cell surface markers, and reporting of immunophenotypes. Additionally, the function(s) of distinct cell types as biomarkers cannot be accurately defined without the proper identification of homogeneous populations. As such, we developed a method for the identification and analysis of human leukocyte populations by the use of eight 10-color flow cytometric protocols in combination with novel software analyses. This method utilizes un-manipulated biological sample preparation that allows for the direct quantitation of leukocytes and non-overlapping immunophenotypes. We specifically designed myeloid protocols that enable us to define distinct phenotypes that include mature monocytes, granulocytes, circulating dendritic cells, immature myeloid cells, and myeloid derived suppressor cells (MDSCs). We also identified CD123 as an additional distinguishing marker for the phenotypic characterization of immature LIN^-^CD33^+^HLA-DR^-^ MDSCs. Our approach permits the comprehensive analysis of all peripheral blood leukocytes and yields data that is highly amenable for standardization across inter-laboratory comparisons for human studies.

## Introduction

Myeloid cells are a diverse group of cells that serve critical roles in the regulation of innate and adaptive immunity. Myeloid cells traffic to sites of injury, internalize and present foreign objects and pathogens, and secret pro- and anti- inflammatory cytokines. In the bone marrow, common myeloid progenitors originate from hematopoietic stem cell progenitors and give rise to granulocytes and monocytes. Monocytes can further differentiate into macrophages and dendritic cells. In addition to regulating normal immune physiology, myeloid cells also participate in regulating both positive and negative responses to tumor formation [1–3].

Our current understanding of myeloid function in normal physiology and disease states, such as tumor development, has largely been derived from murine models with well-defined phenotypes correlating with functions. Similar progress has been slowed in human studies due to the lack of conserved myeloid-derived surface markers on human cells (i.e. human myeloid cells do not express Gr-1) and the overlapping cell surface markers on human myeloid subsets that has made it more difficult to discriminate functional properties by phenotype alone. For example, mature monocytes are typically characterized by the expression of CD33, CD11b, CD14, HLA-DR, and CD16 whereas granulocytes are characterized by CD33, CD11b, CD15, and CD66b. However, CD15 is expressed at low levels on monocytes with some anti-CD15 clones demonstrating variable immunogenicity on monocytes [4, 5]. Conversely, during diseased states, such as sepsis, CD14 expression can be variably expressed by neutrophils [6]. In the case of human myeloid derived suppressor cells (MDSCs), there is considerable diversity in the use of surface markers to define these cells that consequently hinders the ability to accurately measure their function(s). In general, human MDSCs comprise a diverse group of CD33^+^HLA-DR^-^ cells that includes cells granulocytic cells (CD15^+^ or CD66b^+^), monocytes that have lost or diminished HLA-DR expression (CD14^+^HLA-DR^lo/neg^ monocytes or monocytic MDSCs), and immature myeloid cells (Lineage^-^), although many other cell surface markers have been used to identify these cells[7, 8]. The lack of consistent nomenclature and use of cell surface markers creates an untenable situation. For example, monocytic MDSCs can have quite variable expression of CD16. Circulating monocytes have been defined by three subsets by phenotypes with different immunological functions-CD14^+^CD16^-^(classical), CD14^+^CD16^+^(intermediate), and CD14^lo^CD16^+^ (non-classical)[9]. We have additionally seen that these subtypes also differ dramatically in their expression of HLA-DR[10, 11]. Therefore, the designation of monocytic MDSCs is an ambiguous term that often contributes to confusion as to how these cells are described. Finally, circulating dendritic cells (DCs) comprise a diverse group of antigen presenting cells in which phenotypic markers (often first described in animal models) have been assigned to for human DCs; yet it is unknown whether these phenotypic markers specify the activation and/or differentiation state, function, and/or tissue distribution in humans.

Another major obstacle in assessing the changes of human myeloid subsets during healthy and diseased states is the methodology used to process blood samples and the manner in which that data is reported. Density gradient centrifugation, the standard approach to isolate blood mononuclear cells (PBMC), prevents accurate quantification of absolute cell counts and eliminates the granulocyte compartment. Duffy and colleagues demonstrate how three different processing steps (whole blood staining, density gradient purification of mononuclear cells, and the freeze/thaw of mononuclear cells) impact the measurements of MDSCs in a study comparing these cells in patients with gastrointestinal cancer to healthy volunteers[12]. While they found that the increases in the percent of CD14^+^HLA-DR^lo/neg^ monocytic MDSCs were conserved across the three different processes in cancer patients versus controls, the three processes yielded significantly different monocytic MDSC cell counts (cells/μl). We have shown that there is considerable variability in how MDSCs are measured and reported and that reporting MDSC percentages without additional context of the larger “parent” or “grandparent” population can lead to erroneous conclusions[13].

The inconsistent reporting of myeloid and other immunephenotypes in human studies and clinical trials has created significant barriers for comparisons among different studies. To address this, we have developed flow cytometric protocols for measuring non-overlapping phenotypes as cell counts and percentages along with a unique approach to visualize and assess several markers concurrently. Our protocols were developed to evaluate myeloid subsets in particular, but we expanded our protocols to permit concurrent evaluation of lymphocytes as well. Our approach provides much needed clarity on human myeloid cell phenotypes by including surface markers that delineate distinct populations and identify a novel marker, CD123, which may help discriminate immature myeloid cells from other myeloid cells and other MDSCs. The utility of these protocols are demonstrated in a large cohort of healthy volunteers and in a longitudinal study of diffuse large cell B-cell lymphoma (DLBCL) patients. In addition, we show that these protocols can not only be used for peripheral blood analyses, but that they can be used for a variety of biological samples, including bone marrow and pleural effusion. Our methodology permits the quantitation of all myeloid cells (with additional protocols for lymphocytes) as non-overlapping phenotypes and provides a framework for the standardization of human myeloid immunophenotyping.

## Materials and Methods

### Patient samples

Peripheral blood was collected in tubes containing K_2_EDTA as the anticoagulant from 79 healthy controls and cancer patients under Mayo Clinic’s Institutional Review Board approval. Patients with newly diagnosed diffuse large B-cell lymphoma were enrolled in a phase II study and treated with Everolimus (RAD001) and R-CHOP (rituximab, cyclophosphamide, doxorubicin, vincristine, prednisone) (NCT01334502, clinicaltrials.gov). Peripheral blood samples were drawn at baseline, before cycle 3, and 4–6 weeks after the 6^th^ and last cycle of treatment. Peripheral blood (NaHeparin) and pleural fluid (Vacutainer tubes, BD Biosciences, San Jose, CA) were collected from a patient with mesothelioma enrolled in a clinical trial (MC1023). Peripheral blood and bone marrow were from a multiple myeloma patient prior to treatment. All patients provided written informed consent in accordance with the Declaration of Helsinki.

### Flow cytometry of peripheral blood and other biological samples

100 μl of blood, bone marrow, or pleural fluid were added to each tube and blocked for non-specific antibody binding with 50 μl mouse serum (Sigma-Aldrich, St. Louis, MO) at room temperature for 5 minutes. Prior to the addition of the mouse serum, the pleural fluid was concentrated to 1/10 original volume in phosphate buffered saline (PBS) and for the B cell tube, 3 ml of PBS was added, the tube centrifuged, and the supernatant aspirated. Appropriate antibodies (Table 1 and S1 Table.) were added to each tube and incubated in the dark for 15 minutes at room temperature. Red blood cells were lysed with the addition of Versa-Lyse (Beckman Coulter, Indianapolis, IN) for at least 20 minutes at room temperature. For the lyse/no wash assay, 100 μl of Flow-Count Flourospheres (Beckman Coulter) were added, mixed and analyzed. For the lyse/wash tubes, samples were centrifuged, washed in PBS containing 1% albumin and 5mM EDTA, and fixed in 1% paraformaldehyde. Samples were run on the Beckman Coulter Gallios 3-laser, 10-color flow cytometer that was calibrated per manufacturer’s recommendations each day of use. Instrument settings (cytosettings) for each protocol were tailored with unique voltage and compensation matrices. Verify tubes were used to track instrument settings over time. In cases where the antigens are expressed at low levels or do not have clearly defined positive populations, the position of the positive/negative gate was placed based on either different cell populations within the tube that were clearly negative, or the use of a fluorescence minus one (FMO) control tube. List mode data (LMD) files were analyzed using Kaluza software version 1.2 (Beckman Coulter). Leukocyte populations of interest were colored by the representative gate or “back-gated” using histograms of selected stained cell populations. Kaluza software was used to create radar plots. Quantification of leukocytes was calculated by the following formula placed in the Information Table in Kaluza:

**Table 1:** 10-color flow cytometric protocols that enable the quantification of all major leukocyte populations.

Lyse No Wash with FlowCount Panel										
Protocol	FL1	FL2	FL3	FL4	FL5	FL6	FL7	FL8	FL9	FL10
Verify	CD8	CD2	CD20	CD14	CD3	CD7	CD19	CD5	CD4	CD45
TBNK/M/G	CD15	γδTCR	CD16	CD14	CD56	CD19	CD8	CD3	CD4	CD45
**Lyse Wash Panel**										
Verify	CD8	CD2	CD20	CD14	CD3	CD7	CD19	CD5	CD4	CD45
T Cell-1	CCR7	CD27	CD45RO	CD25	CD3	CD62L	CD127	CD45RA	CD4	CD8
T Cell-2	CCR7	CD272	CD45RO	TIM-3	PD-1	CTLA4	CD8	CD28	CD4	CD3
B cell	IgD	CD27	CD20	CD38	CD5	IgM	CD19	CD24	CD21	CD45
Myeloid	LIN2	CD123	HLA-DR	CD11c	CD11b	CD33	CD16	CD66b	CD15	CD45
Monocytes-1	CD80	CD142	CD14	CD32	CD64	CD86		CD16	HLA-DR	CD45
Monocytes-2	B7H1	TNFR2	CD14		PD-1	CD40		CD16	HLA-DR	CD45
Granulocytes	CD66b	CD63	CD14	CD44	CD203c	CCR3	CD16	CD49d	CD15	CD45

Cell count/μl = count(“Phenotype”) X (Flow-Count Flourospheres/μl) /count(“CAL”)

(“CAL” is the total amount of fluorospheres counted during the run time). Where possible, we followed the recommendations of the Minimum Information About a Flow Cytometry Experiment (MiFlowCyt) [14] and outlined in S2 Table.

### Statistical Analyses

All data analysis and graphical representation of the data was performed using Prism 5.0 (GraphPad Software). The non-parametric Mann-Whitney test was used to determine statistical significance of values between HV controls and patients. The Wilcoxon matched-pairs signed rank test was used for assessing differences within time points from the same patients. The non-parametric Spearman test was used to determine significance of correlative data.

## Results

### Quantitation of leukocyte populations in peripheral blood

**Fig 1:**
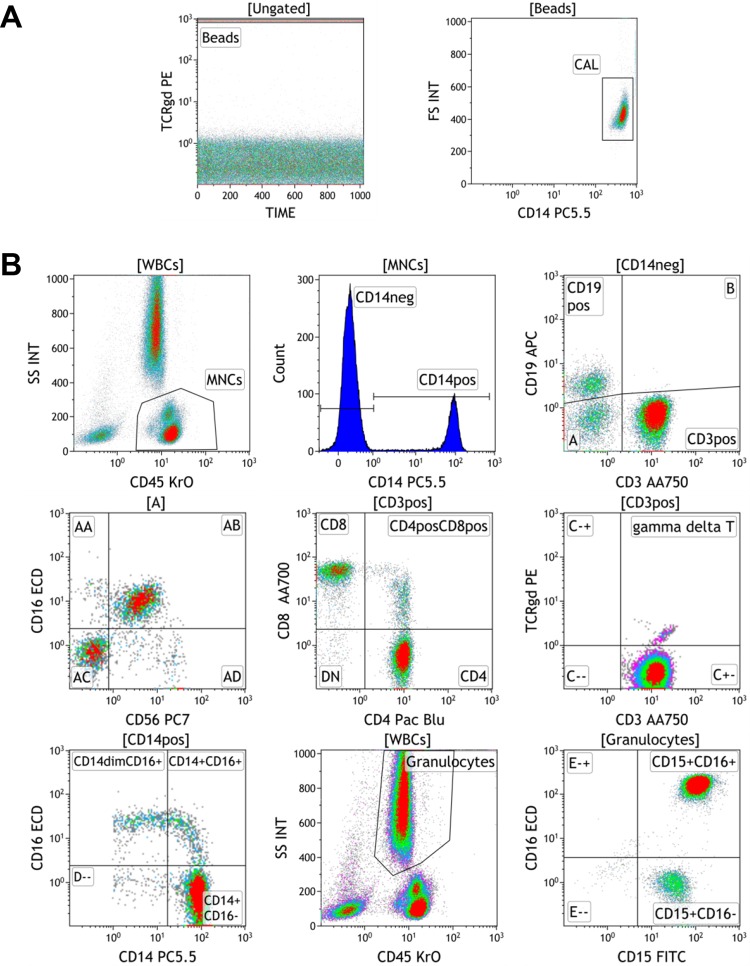
The development of a 10-color protocol to enumerate lymphocytes, monocytes, and granulocytes. Antibodies corresponding to the TBNK/M/G protocol were added directly to fresh peripheral blood. The red blood cells lysed, and run on the Gallios cytometer with the addition of fluorescent counting beads. A. Fluorescent beads are gated on FL2 by time, and total beads collected by FL4 and forward scatter (FS) B. Gating strategy for the enumeration of leukocyte subsets. The gates for the selected populations are listed above each bivariate dot plot or histogram. [WBCs] = total leukocytes gated by forward and side scatter; [MNCs] = mononuclear cells.

In order to standardize our own data for comparisons across multiple studies, we incorporated the use of cell counts to quantify immune cell populations[11]. We have since developed eight 10-color flow cytometric protocols that enable the absolute quantification of all major leukocyte populations (Table 1). Central to the development of these novel 10-color protocols was the ability to quantify leukocyte populations in cell counts using a single platform assay that incorporates the use of fluorescent beads (fluorospheres). Fig. 1 shows our gating strategy for the quantitation of leukocytes in the TBNK/M/G protocol. Fluorospheres have multi-channel detection capacities that we captured by gating on FL2/PE by Time (Fig. 1A). The total amount of fluorospheres was quantified by plotting FL4/PC5.5 by forward scatter. These two sequential gating steps removed the potential for contaminating leukocytes or debris to be included in the fluorosphere population. Phenotypes were enumerated by calculations performed by the Kaluza software analysis program.

Most commercial kits that count lymphocytes are typically 4 or 6 color assays in which CD45, CD3, CD4, CD8, CD19 are used to measure T cells, T cell subsets, and B cells. CD16 and CD56 are often combined in one fluorochrome or channel for the quantitation of NK cells. Since we also wanted a protocol that would also allow us to quantitate monocytes and granulocytes, we separated CD16 and CD56 into different fluorochromes and added CD14 for monocyte detection and CD15 for granulocytes. Finally, we included a pan γδ T cell receptor to capture γδ T cells. These cells have been shown to be involved in a variety of immune functions including anti-tumor immunity and autoimmunity[15]. Blood samples from 79 healthy volunteer controls were stained with the TBNK/M/G protocol (Table 1) and a representative analysis from this cohort is shown in Fig. 1. Leukocytes were first isolated from lysed RBCs by side scatter (SSC) and CD45 (Fig. 1B). Total leukocytes were gated from all CD45 positive cells and gated peripheral blood mononuclear cells (PBMCs) included lymphocytes (low SSC) and monocytes (medium SSC). From PBMCs, CD14^+^ monocytes were removed from the lymphocyte compartment by histogram gating on CD14 positive and negative cells. From the CD14 negative fraction, a bivariate plot of CD19 by CD3 was created to enumerate CD19^+^ B cells, CD3^+^ T cells, and CD3/CD19 negative cells. CD3^+^ cells can be further subdivided into CD4^+^CD8^-^, CD4^+^CD8^+^, CD4^-^CD8^+^, CD4^-^CD8^-^ populations. Although many studies show that γδ T cells lack CD4 and CD8, this protocol allows for potential identification of abnormal expression of γδ TCR on other T cell subtypes. The CD3/CD19 negative lymphocytes can be assessed by CD16 and CD56 to quantitate CD56^+^CD16^+^ and CD56^+^CD16^-^ NK cells, CD16^+^CD56^-^ cells, and lineage negative cells that are CD14^-^CD3^-^CD19^-^CD16^-^CD56^-^. By using a separate designated fluorochrome for CD16 antibody, subpopulations of monocytes and granulocytes can be enumerated. Monocyte subsets are gated on a CD14 by CD16 bivariate plot of the CD14^+^ cells gated from PBMCs. Granulocytes gated by high side scatter were assessed for CD15 and CD16 to enumerate neutrophils (CD15^+^CD16^+^) and eosinophils (CD15^+^CD16-). Overall, the combination of these 10 markers yields, at minimum, 20 distinct, non-overlapping phenotypes that can be reported as cell counts, percentages of parent and grandparent populations, and even ratios of one cell type to another (i.e. CD4/CD8 ratio) when staining whole blood. The mean, standard deviation, and ranges for each phenotype are listed in S3 Table.

### Novel analyses of leukocyte subsets

**Fig 2:**
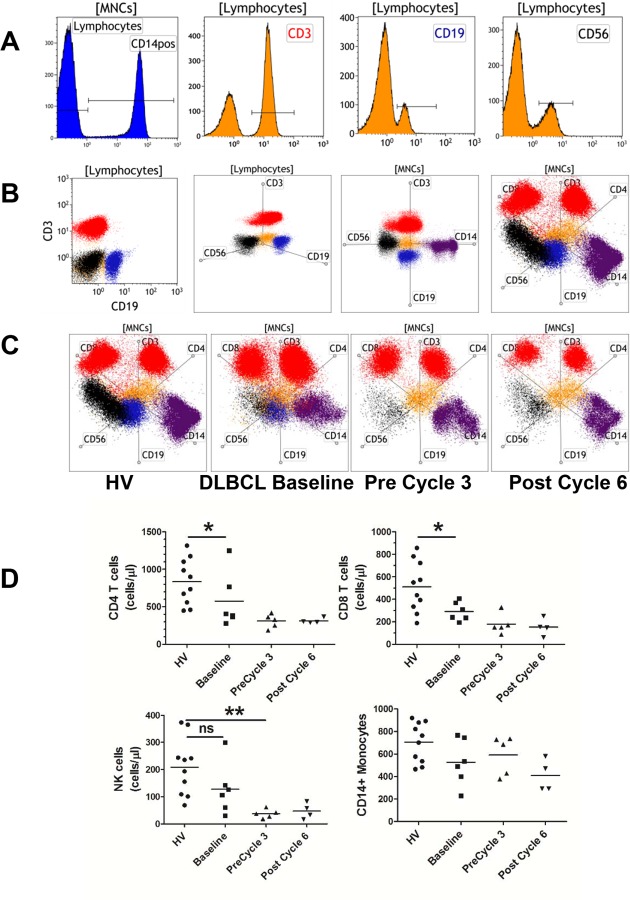
Radar plots permit simultaneous visualization of leukocyte compartments. Fresh blood was stained with the TBNK/M/G protocol and analyzed per Fig. 1. A. Gated populations from histograms for selected markers were color coded for radar plot analyses. Lymphocytes (orange) and CD14pos (purple) were gated from mononuclear cells ([MNCs]). CD3 (red), CD19 (blue), and CD56 (black) were gated from the Lymphocyte gate. B. Bivariate dot plot and radar plot comparisons. Populations were either gated from the MNC or Lymphocyte gate. Radar plots show populations on 3, 4, and 6 axes. The markers for the 6 axes include CD3, CD4, CD8, CD14, CD19, and CD56. C. Radar plot analyses of longitudinal data from a patient and a healthy control. The arrangement of the 6 axes in each plot is identical for each sample. D. Graphical representations of the longitudinal cell count data collected from 5 DLBCL patients and 10 healthy volunteer controls (HV). * = *P* value < 0.05.

Our TBNK/MG/ protocol enables the quantitation of monocytes and granulocytes in addition to the lymphocyte population. With the substantial increase in the amount of measured phenotypes from this protocol over previously developed antibody combinations, we sought new ways of assessing and interpreting the data that will enable us to apply the same gating strategies to multiple control and patient samples. The radar plot analysis tool in the Kaluza software allows rapid visualization of the distribution of leukocytes of interest. We also took advantage of “back-gating” of parent populations and color-coding each population of interest. Using data from a healthy volunteer, we gated CD14+ monocytes from total mononuclear cells by a histogram of CD14 expression. The CD14- cells contain a mixture of lymphocytes and immature cells. For simplicity, we denoted the CD14- gate “Lymphocytes” (colored orange) and the CD14+ gate “CD14pos” (colored purple) (Fig. 2A). From the Lymphocyte gate, we gated T cells (CD3, red), B cells (CD19, blue), and NK cells (CD56, black) (Fig. 2A). Traditionally, two markers of interest on a particular cell type are plotted on a bivariate dot plot. For example, we plotted CD19 versus CD3 from cells gated from the Lymphocyte population (Fig. 2B). The radar plot enables simultaneous examine of the entire lymphocyte population. Thirteen parameters can be added to a radar plot as independent axis. The position of a particular population on each axis is similar to the placement on dot plots where the higher expressing/brighter markers will be farther away from the center and the negative/dim markers will be close to the center. We started with three markers, CD3, CD19, CD56, and arranged the axes similar to a typical x,y,z three dimensional plot (Fig. 2B, second graph). Here the additional axis efficiently separates the NK cells (black) from Lineage negative cells (orange) and B cells (blue) unlike the bivariate plot (Fig. 2B). Next we changed the input gate from Lymphocytes to mononuclear cells (MNCs) and added CD14 (purple) (Fig. 2B, third graph). Axes were added sequentially to determine how the populations shift when an additional marker is added. Finally, we added CD4 and CD8 to further separate the CD3 population (red) (Fig. 2B, last graph). S1 Video shows how these populations move in relation to each other when the axes are rotated. We found that since certain markers stained unrelated cells (i.e. CD8 is expressed strongly on a subset of T cells but is also expressed on NK cells while CD4 is dimly expressed on monocytes in addition to strong expression on T cells), some populations were influenced by multiple axes. Additionally, when populations are strongly positive for two markers in which the axes are 180 degrees apart, they will appear negative for both. Therefore, depending on the markers selected for this type of analysis, the arrangement of the axes must be empirically determined to find the most optimal configuration of markers. In Kaluza, the analysis can be saved as a template and applied identically to other data files collected under the same instrument settings.

We envision that the best use for radar plots is for longitudinal studies in which patients can be assessed over time with or without interventions. We tested the usefulness of this strategy in a subset of newly diagnosed diffuse large B cell non-Hodgkin lymphoma (DLBCL) (n = 5). These patients were enrolled in a Phase II clinical trial where they received treatment with six cycles of everolimus (RAD001) and R-CHOP (rituximab, cyclophosphamide, doxorubicin, vincristine, prednisone) (NCT01334502). Blood samples were drawn at baseline prior to treatment, before 3^rd^ cycle of therapy, and 4 to 6 weeks after completion of therapy, at the time of assessment for clinical response. These samples along with blood samples from healthy controls were analyzed using the TBNK/MG protocol as outlined in Fig. 1 and Fig. 2B. Fig. 2C shows a longitudinal radar plot analysis from one patient compared to a control outlined in Fig. 2B. After chemoimmunotherapy, lymphoma patients had a near complete loss of B cells, as expected, along with reductions in CD4 and CD8 T cells but no effect on monocyte counts. Interestingly, the drop in total leukocytes (1368 cells/μl at baseline to 846 cells/μl at post-cycle 6 resulted in the percentage of monocytes of the mononuclear compartment to rise from 14% at baseline to 35% at the post-cycle 6 treatment. The blood of an additional 4 DLBCL patients were similarly analyzed and showed the same trajectory (Fig. 2D). Our data demonstrated that these 5 patients had lower CD4 and CD8 counts prior to treatment than healthy controls, suggesting disease specific changes. Overall, the combination of our TBNK protocol and radar plot analyses provide a novel tool to rapidly enumerate and visualize immunophenotypic changes suitable for human studies.

**Fig 3:**
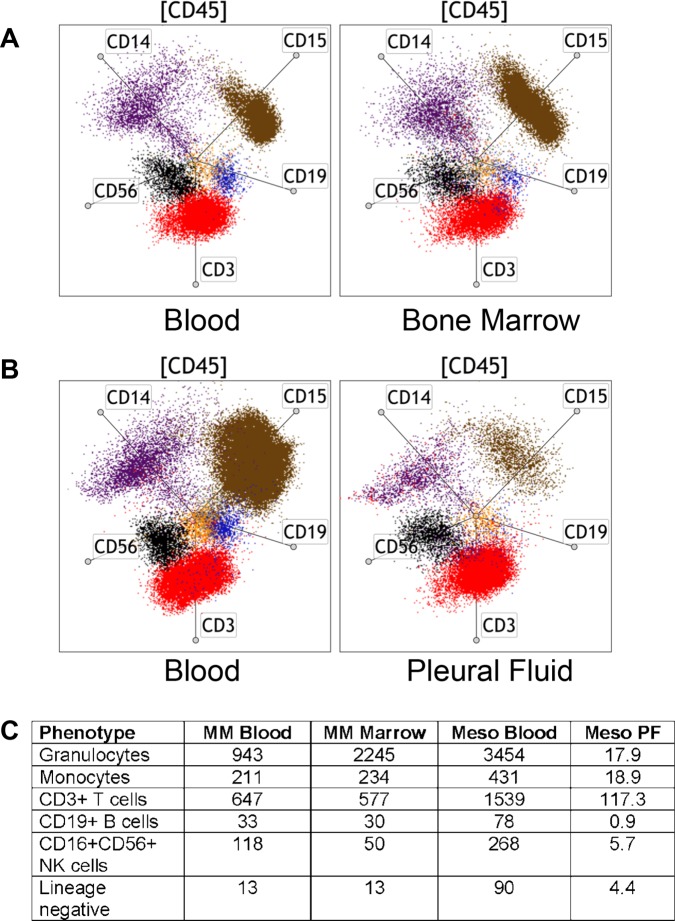
The TBNK/M/G protocol and radar plot analysis are ideal for comparisons of different biological samples. Radar plot analyses on (A) peripheral blood and bone marrow samples from a multiple myeloma patient and (B) peripheral blood and pleural fluid from a mesothelioma patient. The radar plot configuration includes CD3 (red), CD19 (blue), CD56 (black), CD14 (purple), and CD15 (granulocytes, brown) gated from total CD45+ cells ([CD45]) and the axes are identical across all samples. (C) Summary of cell count data from each of the examples.

In addition to the utility of radar plot analysis in longitudinal studies, the radar plot allows rapid visualization of multiple phenotypes across biological examples from the same individual. We tested the ability to determine the distribution of leukocytes in peripheral blood and bone marrow from multiple myeloma patients. We stained blood and bone marrow similarly with the TBNK/M/G protocol and performed the analysis as shown in Fig. 1 to obtain cell counts. Additionally, we show in a radar plot the data from CD45^+^ cells in one patient (Fig. 3A). The radar plot from this patient reveals that the bone marrow has a modest change in distribution of leukocytes that is reflected by a reduction in T cells and NK cells compared to the blood. From the cell count data, we see this change is due to a large increase in the number of granulocytes in the marrow (943 cells/μl in blood versus 2245 cells/μl in the marrow). In another example, we examined the changes in leukocyte distribution in peripheral blood and pleural fluid of mesothelioma patients. Dramatic differences in the distribution of leukocytes are easily observed between peripheral blood and pleural fluid. In this example, granulocytes are the major population in peripheral blood (59%), whereas T cells are the most prominent cell type in pleural fluid from the same patient (71%) (Fig. 3B). The cell count data for the phenotypes shown in the radar plots are shown in Fig. 3C. These examples demonstrate the significant potential for our methodology to accelerate novel discoveries and the development of hypothesis driven research in human studies and clinical trials.

### Myeloid phenotypes

**Fig 4:**
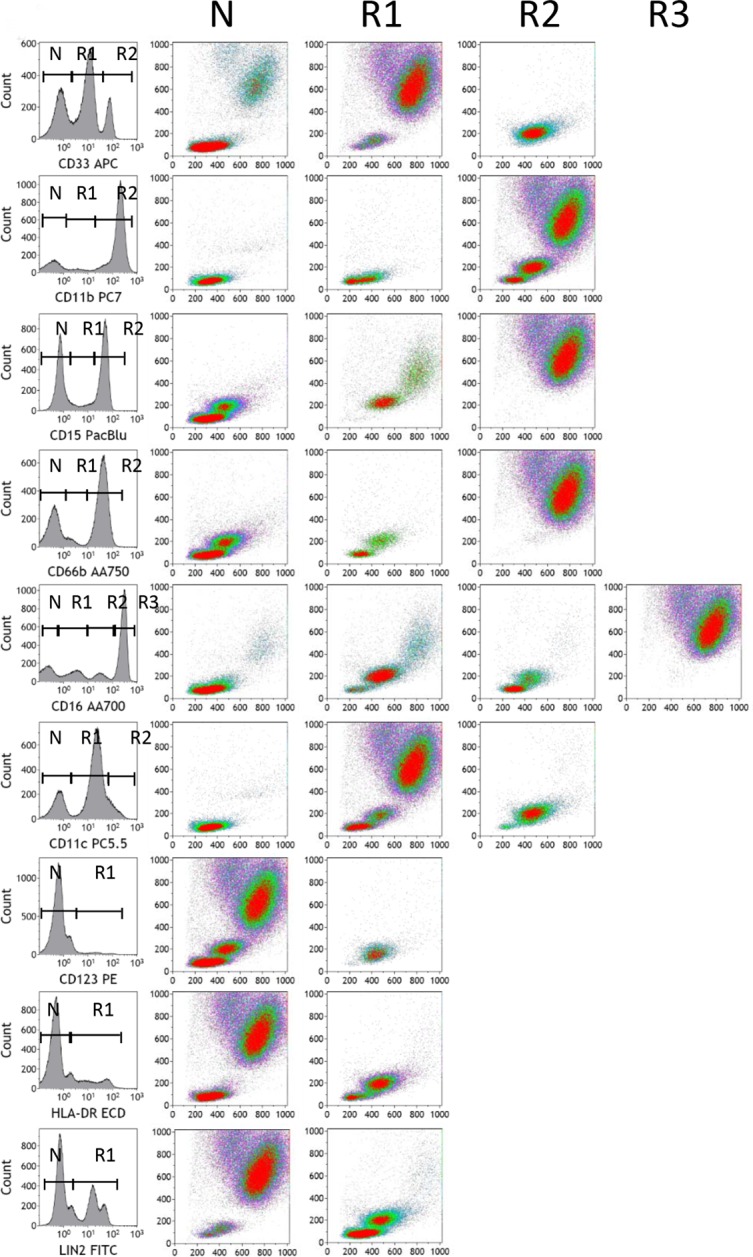
Characterization of myeloid marker staining patterns on whole blood. Peripheral blood from a healthy control sample was processed via the lyse/wash Myeloid protocol. Histograms were generated from each of 9 antibodies used to delineate myeloid populations. In most cases regions (R1, R2, and R3) were created for each peak of expression including peaks with no expression (N). Other regions represent populations of cells falling between the negative/positive peaks or populations that do not have a clear peak. Density gradient plots were created from cells gated from each region plotted by forward and side scatter. A representative example from a healthy volunteer is shown.

We have previously shown that CD14^+^HLA-DR^lo/neg^ monocytes negatively impact the outcome of cancer patients[16–18]. In order to consistently report and compare myeloid immunophenotypes across studies, we defined non-overlapping myeloid phenotypes using 10 distinct fluorochrome markers in a single analysis. We started with myeloid phenotypes that have either been traditionally assigned to cell types or are markers of function. Our myeloid protocol was designed to account for all unique populations of myeloid cells including human MDSC populations. In order to understand the performance of the antibodies we selected for analysis, we created histograms for 9 of the 10 myeloid markers (excluding CD45) from staining whole blood from a healthy controls and created histogram gates for peaks/regions representing negative (N), positive (R1) and/or strongly positive (R2 and/or R3) populations. Cells from each gate were then plotted to determine their positions by forward and side scatter parameters (Fig. 4). This type of analysis revealed the distribution of positive and negative cells within the same sample. For example, CD33 demonstrated three peaks in which strongly positive cells (R2) were all monocytes, moderately positive cells (R1) were granulocytes and cells that fall in between typical lymphocyte and monocyte forward/side scatter positioning. Interestingly, CD11b staining was less specific for myeloid cells in that some lymphocytes were strongly positive for CD11b (R2). Granulocytes were strongly positive for CD15 (R2) and CD66b (R2), although a fraction of monocytes showed low expression of CD15 (R1) and CD66b (R1). Granulocytes and monocytes also showed distinguishing expression of CD16 (gate R3 vs. R1 and R2), CD11c (gate R1 vs. R2), and HLA-DR (R1 vs. R2). Finally, CD123^+^ cells were not granulocytes or mature monocytes, and didn’t fall within typical lymphocyte forward/side scatter properties.

**Fig 5:**
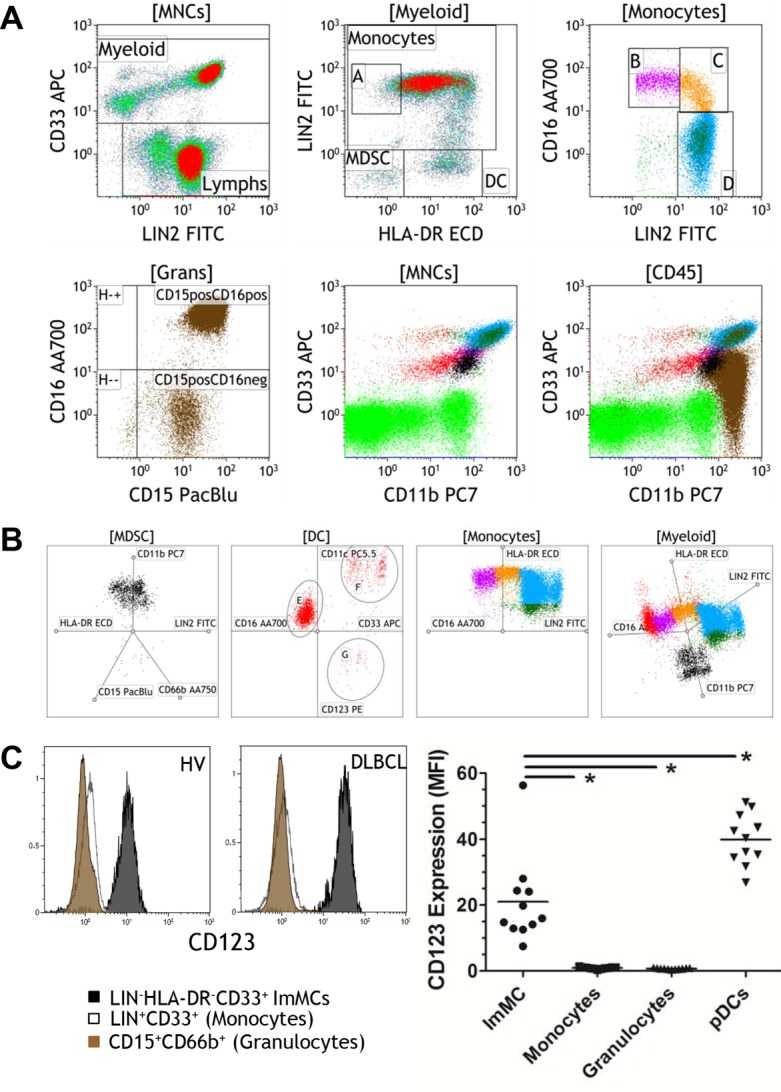
10-color analysis of myeloid cells reveals complexity of myeloid phenotypes. Peripheral blood from a healthy control sample was processed via the lyse/wash Myeloid protocol. A. Bivariate plots were used to delineate and color-code myeloid populations. CD33 was used to distinguish myeloid cells from lymphoid cells. LIN2 was also used as a surrogate for CD14. A plot of LIN2 and HLA-DR from the Myeloid gate was used to further separate cells into mature monocytes (LIN2+, [Monocytes]), LIN2+HLA-DR^lo/neg^ monocytes ([A] in dark green), LIN2-CD33+HLA-DR- myeloid derived suppressor cells (MDSC in black), and circulating dendritic cells (DC in red). Monocytes were further gated into classical monocytes LIN2+CD16- (blue), intermediate monocytes (orange), and non-classical monocytes (purple). Granulocytes were gated from CD45+ high side scatter populations and colored brown. CD33 by CD11b dot plots from mononuclear cells ([MNCs]) and total leukocytes ([CD45]) using the same color coding scheme listed above. B. Radar plots of myeloid cells were generated from MDSCs, DC, Monocytes and total myeloid cells ([Myeloid]). Selected markers and axes were arranged uniquely for each myeloid subset. C. Histograms showing representative examples of CD123 expression on LIN^-^HLA-DR^-^CD33^+^ ImMC MDSC (black), LIN^+^CD33^+^ monocytes (white), and CD15^+^CD66b^+^ granulocytes (brown) from a healthy volunteer and DLBCL patient. The graph shows the geometric mean fluorescence intensity of CD123 on LIN^-^HLA-DR^-^CD33^+^ MDSC (ImMC), LIN^+^CD33^+^ monocytes (white), and CD15^+^CD66b^+^ granulocytes and CD123^+^CD11c^-^ plasmacytoid dendritic cells (pDC) from healthy volunteers (n = 11). * = p<0.05 as determined by two-tailed Wilcoxon matched-pairs signed rank test.

We developed our gating strategies of myeloid populations with the understanding of the staining characteristics of the antibodies shown in Fig. 4 and in combination with traditional gating strategies. Using healthy volunteer blood samples, myeloid cells were separated from other mononuclear cells by a LIN2 and CD33 dot plot (Fig. 5A: upper left plot). LIN2 contains lineage markers CD3/CD14/CD19/CD56 and does not include CD16, an important marker for delineating myeloid subsets. CD16 was used in its own fluorochrome resulting in greater specificity to identify myeloid populations. The CD33^+^ myeloid cells in the mononuclear compartment can further be defined by LIN2 and HLA-DR. In this plot, CD33^+^LIN2^+^ are mature monocytes that are CD14^+^. LIN2^+^(CD14^+^) monocytes that have low or no HLA-DR expression (Fig. 5A upper middle plot; gate A) are CD14^+^HLA-DR^lo/neg^ monocytes or monocytic MDSCs. LIN2^neg^HLA-DR^+^ cells are circulating dendritic cells (DC gate, upper middle plot; Fig. 5A) and LIN2^neg^HLA-DR^neg^ cells include both immature MDSCs and granulocytes/granulocytic MDSCs as as previously defined[19, 20] (MDSC gate, middle panel; Fig. 5A). Monocytes were further subgrouped into non-classical (Gate B, upper right plot, Fig. 5A), intermediate (Gate C, upper right plot, Fig. 5A), and classical (Gate D, upper right plot, Fig. 5A) monocytes. Other myeloid cells including granulocytes are identified by high side scatter and CD15^+/-^ CD16 expression (Fig. 5A, lower left plot). Often CD11b and CD33 are used to identify myeloid cells and, more specifically, MDSCs. Mature monocytes demonstrate high expression of CD33 and CD11b and granulocytes exhibit moderate CD33 positivity and high CD11b expression. LIN2^neg^ cells demonstrate varying degrees of CD33 and CD11b expression. By color coding each myeloid gate we can determine the degree of CD33 and CD11b expression on each cell type. We observed considerable overlap of distinct populations when we plotted CD33 by CD11b on mononuclear cells (Fig. 5A; lower middle plot). DCs (red) and MDSCs (black) showed nearly identical expression of CD33 and CD11b. When gating all leukocytes (CD45 gate), a significant portion of granulocytes (brown) also overlapped with DCs and MDSCs (Fig. 5A; lower right plot).

The use of additional markers and new methods of analysis will help to further define myeloid populations. Using the radar plot analysis for LIN2^-^CD33^+^HLA-DR^-^ MDSCs, we observed that they are CD11b^+^CD15^-^CD66b^-^ (Fig. 5B; [MDSC]). For DCs, using CD11c, CD33, CD123, and CD16 for radar plot analysis, there were three major DC populations: CD33^+^CD11c^+^CD16^-^CD123^-^ myeloid DCs, CD33^+^CD11c+CD16-CD123^+^ DCs, and CD33^+^CD11c^+^CD16^+^CD123^dim^ DCs (Fig. 5B; [DC]). The myeloid DCs likely include BDCA1 and BDCA2 DC populations (data not shown) and the CD16^+^ DCs may represent monocytes with DC-like properties[21]. The CD33^+^CD123^+^CD11c^-^ DCs may be a subpopulation of plasmacytoid DCs as CD123^+^ cells have been shown to have some CD33 expression[22, 23]. For mature monocytes, the radar plot analysis demonstrates the variation in HLA-DR expression among the three subtypes of monocytes (Fig. 5B; [Monocytes]). When the myeloid population is plotted with 4 markers (LIN2/HLA-DR/CD16/CD11b) on a radar plot, a continuous spectrum of CD16 expression is readily observed and suggestive of an inverse relationship between LIN2/CD14 and CD16 (Fig. 5B; [Myeloid]). As we have shown with the TBNK/M/G protocol, we animated the axes to show how these populations move in relation to each other (S2 Video). Overall, these results highlight the diversity of myeloid phenotypes and the requirements of multiple markers to accurately define each unique phenotype.

The careful characterization of the staining patterns of the antibodies used to identify myeloid populations and the use of multi-dimensional analyses (radar plots) to look at the myeloid population as a whole allows us to better understand the changes in cell populations that occur in cancer, autoimmunity, and infection. The ability to measure 10 markers simultaneously greatly increases number of analyzable phenotypes. We list several phenotypes in S4 Table with expression indicated by regions outlined in Fig. 4. Defining surface expression of each marker in this manner allows for a precise description of each phenotype. For example, CD33^+^HLA-DR^-^ cells are commonly reported as MDSCs with variable and/or inconsistent expression of CD15. Our data suggests that there are multiple cell types that can be described as CD33^+^HLA-DR^-^: Granulocytes (SS^hi^CD33^R1^CD15^R2^CD66b^R2^HLA-DR^N^), monocytes that have lost HLA-DR expression (SS^med^CD33^R2^LIN2^R2^HLA-DR^N^), and immature myeloid cells (SS^lo^LIN2^N^CD33^R1^HLA-DR^N^) can all be defined as CD33^+^HLA-DR^-^. The phenotypic data shown here from whole blood are similar to the observations and gating strategies measured from fractionated samples as described by Dumitru et al.[24]. Each of these cell types is distinct in that they clearly have different forward and side scatter properties. SS^lo^LIN2^N^CD33^R1^HLA-DR^N^ immature myeloid cells were clearly negative for granulocytic markers CD15 and CD66b in healthy volunteers and in DLBCL patients. However, we found that these cells express high levels of CD123/IL3-R whereas granulocytes and monocytes were clearly negative for this marker (Fig. 5C). CD123 levels were about half the levels of plasmacytoid DCs but 20 fold higher than monocytes and granulocytes. In this analysis, SS^lo^LIN2^N^CD33^R1^HLA-DR^N^ cells averaged nearly 96% positive for the CD123 marker (range 92.1–98.4%; n = 11 healthy volunteers). A comparison of staining patterns for SS^lo^LIN2^N^CD33^R1^HLA-DR^N^ cells and plasmacytoid DCs (which are CD123 bright) are shown in S1 Fig. As basophils have also been shown to express CD123[25], and can also be defined as LIN^-^CD33^+^HLA-DR^-^, we examined the amount of two additional markers that stain for basophils, CD15^+^[26]and CD16b^+^[27], in SS^lo^LIN2^N^CD33^R1^HLA-DR^N^ gated cells. From the same healthy volunteers analyzed for CD123, approximately 2.5% of cells were CD15^+^CD123^+^ positive (0.07%- 16.6%) and about 1.8% CD15^+^CD16b^+^ (0.8%- 3.5%) indicated that SS^lo^LIN2^N^CD33^R1^HLA-DR^N^ cells include a small fraction of basophils (S1 Fig.). These findings may help clarify the immunophenotypic characterization of the diverse population of human MDSCs.

From the data and gating strategies outlined in Figs. 4 and 5, we summarized the most common myeloid phenotypes with their associated nomenclature by our non-overlapping phenotypic analysis (Table 2). MDSCs are also shown with nomenclature suggested by two different reviews of MDSCs[8, 24] as well as alternative designations for monocytic MDSCs[18, 28, 29]. This phenotypic analysis serves as a starting point from which to assign and assess function from well-defined populations. With the use of unfractionated whole blood, additional myeloid data can be measured in ways that are lost when cells are purified by density gradient fractionations. Consequently, in addition to phenotype percentages from a fraction of cells (mononuclear cells), each phenotype can be measured as a percent of the total myeloid cells (CD33), total leukocytes (CD45), and cell counts (in combination with the TBNK/M/G protocol).

**Table 2:** Phenotypic characterization of the most common myeloid subsets.

	Phenotype	Common Nomenclature	Nomenclature (Ref. [8])	Nomenclature (Ref. [24])	Immune Function
CM1	SS^med^CD33^++^CD11b^++^CD11c^++^CD14^+^CD16^-^HLA-DR^+^CD15^-^CD66b^-^CD123^-^	Classical monocytes			Mixed
CM2	SS^med^CD33^++^CD11b^++^CD11c^++^CD14^+^CD16^+^HLA-DR^+^CD15^-^CD66b^-^CD123^-^	Intermediate monocytes			Positive
CM3	SS^med^CD33^++^CD11b^++^CD11c^++^CD14^lo^CD16^+^HLA-DR^+^CD15^-^CD66b^-^CD123^-^	Non-classical monocytes			Positive (killer myeloid cells)
CM4^1^	SS^med^CD33^++^CD11b^++^CD11c^++^CD14^+^CD16^-/+^ HLA-DR^lo/neg^ CD15^-^CD66b^-^CD123^-^	Suppressive Monocytes^2^	MO-MDSC	MO-MDSC	Suppressive
CM5	SS^lo^CD33^++^CD11b^+/-^CD11c^++^CD14^-^CD16^-^HLA-DR^+^CD15^-^CD66b^-^CD123^-^	Monocytic dendritic cells			Positive
CM6	SS^lo^CD33^+^CD11b^+/-^CD11c^++^CD14^-^CD16^+^HLA-DR^+^CD15^-^CD66b^-^CD123^-^	CD16+ dendritic cells			Positive
CM7	SS^lo^CD33^+^CD11b^++^CD11c^+^CD14^-^CD16^-^HLA-DR^-^CD15^-^CD66b^-^CD123^+^	Immature myeloid cells and/or MDSCs^3^		ImMC	Suppressive
CM8	SS^hi^CD33^+^CD11b^++^CD11c^+^CD14^-^CD16^+^HLA-DR^-^CD15^+^CD66b^+^CD123^-^	Granulocytes/ neutrophils	GR-MDSC^4^	GR-MDSC	Mixed
CM9	SS^hi^CD33^+^CD11b^++^CD11c^+^CD14^-^CD16^-^HLA-DR^-^CD15^+^CD66b^+^CD123^-^	Granulocytes/ eosinophils	GR-MDSC^4^	GR-MDSC	Mixed

^1^: This is a subset of CM1, CM2 and CM3 where DR has been lost. Often this designation is currently grouped independent of the designation of CD16. However, CM1, CM2 and CM3 have been shown to have unique positive effects on immunity. Thus, an alternative designation for the suppressive version of CM1, CM2 and CM3 is CM1-DRneg, etc.

^2^: Termed suppressive monocytes (Refs. [17, 18, 28, 29]).

^3^: MDSC were only classified as MO-MDSC and GR-MDSC without clear designation of what these cells and/or subsets are^8^.

^4^: GR-MDSCs generally have the same surface markers as granulocytes but are identified by altered density properties in density gradient purifications of mononuclear cells.

**Fig 6:**
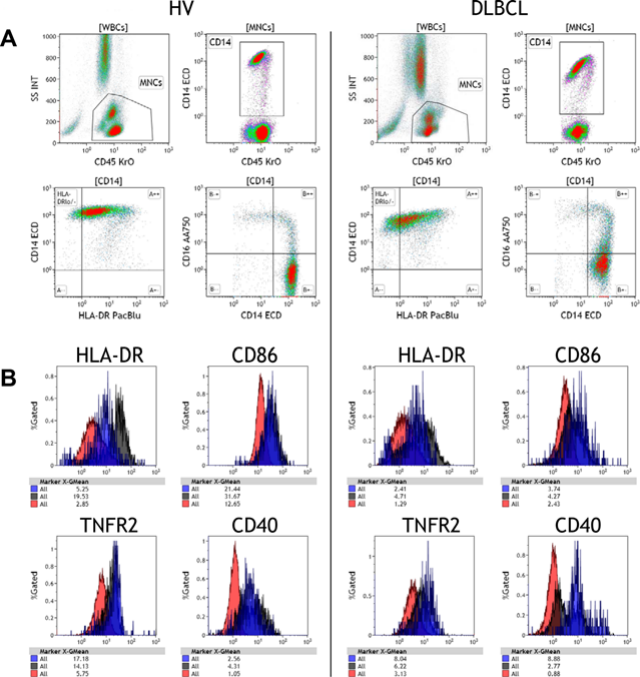
Additional analyses of monocytes reveals diversity of surface expression on classical, intermediate, and non-classical monocytes. A. Examples of monocyte gating from a healthy volunteer control and DLBCL patient. Mononuclear cells were gated from CD45+ WBCs ([WBCs]). CD14^+^ monocytes were gated from [MNCs]. CD14^+^ cells were either plotted with HLA-DR to identify HLA-DR^lo/neg^ populations or CD16 to delineate CD14^+^CD16^-^ classical monocytes, CD14^+^CD16^+^ intermediate monocytes, and CD14^lo^CD16^+^ non-classical monocytes. B. Histogram overlays of HLA-DR, CD86, TNFR2, and CD40 on a healthy volunteer control and DLBCL patient. Classical monocytes were colored in red, intermediate monocytes colored in black, and non-classical monocytes were colored blue.

The Monocyte-1 and Monocyte-2 protocols were developed as complementary protocols to the Myeloid protocol in order to further delineate mature monocytes. The inclusion of essential markers like CD45, CD14, CD16, and HLA-DR allow for straightforward analysis of monocyte subsets and CD14^+^HLA-DR^lo/neg^ immunosuppressive population. The other six channels allow for the addition of markers of interest. For these protocol we put B7 molecules, CD32, CD64, and Tissue Factor (CD142) in the Monocyte-1 protocol, and a combination of immunosuppressive molecules (B7-H1 and PD-1), and pro-inflammatory molecules (TNFR2, CD40) in the Monocyte-2 panel. We chose these markers based on our previously published studies[10, 11, 17, 18] but they can be substituted easily to tailor the protocols to diverse patient cohorts. We tested the utility of these two protocols by testing a cohort of untreated diffuse large B cell lymphoma patients (DLBCL) compared to that of healthy controls. Fig. 6A shows the gating strategies of the Monocyte protocols with representative samples from a healthy volunteer and a DLBCL patient. We observed changes in the distribution of monocytes and an increase of CD14^+^HLA-DR^lo/neg^ monocytes in the DLBCL patient. Using the Kaluza software, we gated cells from the quadrants in the bivariate plots of CD14/CD16 (Fig. 6A), and then measured the expression of HLA-DR, CD86, TNFRII, and CD40 for HVs and DLBCL patients. The DLBCL patient exhibited dramatic reductions in HLA-DR, CD86, and in TNFR2 expression on all three monocyte subsets, but had elevated CD40 expression on non-classical monocytes (Fig. 6B). The data shown here provide examples of how these protocols can be used to dissect the potential changes in a patient population.

**Fig 7:**
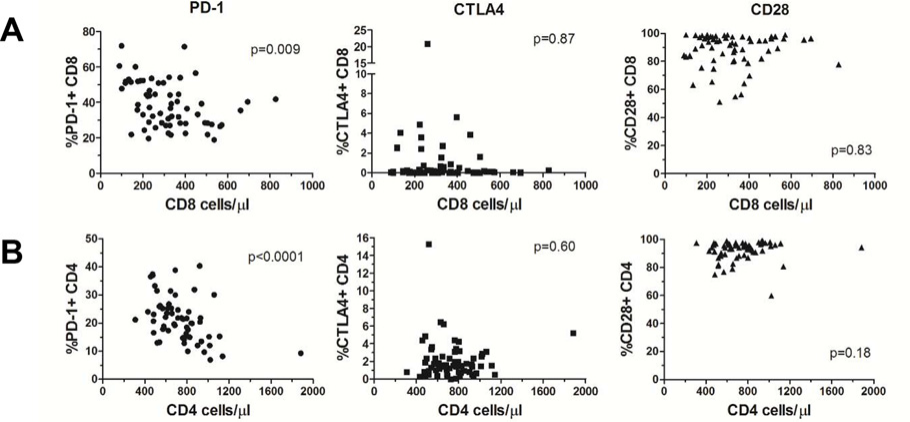
Identification of potential mechanistic associations of immune phenotypes using the combination of relational data and cell counts. Immune phenotype values were measured using the TBNK and T cell-2 protocols from 64 healthy volunteers. The percentages of PD-1, CTLA4, and CD28 positive CD8 or CD4 cells were plotted against either total (A) CD8 cells/ml and (B) CD4 cells/ml. Populations were tested for statistical significance using the Spearman correlation test.

While we focused on creating flow protocols to define human myeloid populations, we also developed additional protocols to perform the same types of analyses on T cells, B cells, and granulocytes. These protocols with the suggested gating strategies are listed in Table 1 and S2 Fig., S3 Fig., S4 Fig., and S5 Fig. These protocols provide a new avenue to build a more complete picture of the immune system as a whole in order to help us understand how the system changes in a particular disease and corresponding treatment. The combination of collecting cell count data in addition to data measured as percentages of a group provides new opportunities for discovery and cultivates novel hypothesis driven questions. To illustrate this, we phenotyped 64 healthy individuals with the TBNK and the T cell-2 protocol (S2 Fig.) and compared the percentages of positive (CD28) and negative (PD-1 and CTLA4) co-stimulatory molecules on CD4 and CD8 cells (obtained from the T cell-2 protocol) to the total CD4 and CD8 cell counts (measured from the TBNK protocol). Interestingly we found a very strong relationship between the amount of CD8 cells/μl and PD-1 levels (p = 0.009; Fig. 7A). Whereas high CD8 cells/μl correlated with low PD-1+ percentages of CD8 cells, we found no such correlation with either CTLA4 or CD28. This finding held true for CD4 cells, with even a stronger association of low PD-1+CD4 and high CD4 cells/μl (p<0.0001; Fig. 7B). These results provide intriguing observations that PD-1 expression associates with circulating CD4 and CD8 levels in peripheral blood and suggests a potential mechanistic role of PD-1 in T cell homeostasis. This data provides a highly relevant example demonstrating how measuring cell counts can reveal new insights into human biology.

## Discussion

Advances in flow cytometry technologies are revealing greater complexities of the human immune system in disease. Therefore, the harmonization of sample preparations, phenotypic markers, gating strategies, data analysis, and presentation is absolutely critical for understanding normal immune homeostasis, disease related changes, and responses (or lack thereof) to immunotherapies. In an attempt to address these issues, we developed an 8-tube protocol utilizing up to ten colors that permits the detection and analysis of all major leukocytes. These protocols permit the direct measurements of cell counts (single platform) as well as the ability to calculate cell counts calculations of cell counts from lyse/wash samples using staining protocols on whole blood samples. We incorporated commonly used phenotypic markers and straightforward gating strategies that permit the enumeration of distinct, non-overlapped phenotypes. In addition to traditional ways of presenting flow data (i.e. histograms and dot plots), we used radar plots in order to simultaneously visualize all leukocytes of interest on one plot. This type of analysis allows for rapid identification of the changes of the distribution of leukocytes between individuals, longitudinally from the same individual, or different biological samples from the same individual. By exploiting the use of 10-colors and the radar plot analysis we were able to discriminate between myeloid populations more precisely.

Our primary interest is the characterization of human myeloid cells and MDSCs. MDSCs have generated great interest due to their role in tumor-mediated immune suppression. Until now, the nomenclature and phenotypic characterization of human MDSCs has been particularly inconsistent. Our myeloid protocol provides clarity to the phenotypic characterization of myeloid cells in that the 10-color approach allows for much improved delineation of myeloid subsets. Our data from healthy controls and patients with DLBCL clearly show that CD14^+^HLA-DR^lo/neg^ monocytes are distinct from immature LIN^-^CD33^+^HLA-DR^-^ MDSCs. The LIN^-^CD33^+^HLA-DR^-^ MDSCs, are in turn, not mature, activated, or degranulated granulocytes (as determined by the lack of CD15 and CD66b expression). LIN^-^CD33^+^HLA-DR^-^ cells are immature myeloid cells as described by Almand et al[19]. Current descriptions of granulocytic MDSCs rely on their capacity to escape density gradient purification from mononuclear cells and our protocols performed on whole blood cannot distinguish granulocytes from granulocytic MDSCs with the available cell surface markers. Our data was collected from unprocessed samples to reduce artifact from sample handling. Since granulocytes from healthy individuals are CD33^+^CD11b^+^HLA-DR^-^ and do express Arginase-I[30], additional phenotypic markers and/or functional assays are needed to further define granulocytic MDSCs. Moreover, our data demonstrates that reliance on CD33 and CD11b alone as markers to define MDSCs is inaccurate and should be avoided due to the significant overlap of these markers myeloid subsets. We identified that CD123 was highly expressed on immature LIN^-^CD33^+^HLA-DR^-^ cells and was not expressed on granulocytes or monocytes. Future studies on the role of CD123 as a definitive marker of immature myeloid cells/MDSCs are justified.

## Conclusions

The data generated from our protocols will permit detection of well over 120 immunophenotypes but could potentially reach 2.9 x 10^6^ (9! x 8 for each protocol) for CD45+ cells. The magnitude of this data will be ideally suited for bioinformatic analyses across disease spectrums as well as across multi-institutional clinical trials. Our aim is to use the rationally designed methodologies outlined here to build a comprehensive immune database of healthy and disease-specific states in order to better understand the dynamics of disease-induced immune alterations. This data can also be used to help inform immune based therapeutic strategies. This methodology provides a framework for the standardization of human immunophenotypes and may enable new insights into human immunology and accelerate hypothesis driven research in human studies and clinical trials.

## Supporting Information

S1 Fig Additional cell surface characterization of LIN^-^CD33^+^HLA-DR^-^ imMC MDSCs. LIN^-^CD33^+^HLA-DR^-^ imMC MDSCs. (black) were displayed on the same dot plots as LIN^-^CD123^+^CD11c^+^HLA-DR^+^ imMC plasmacytoid dendritic cells (red) for comparison of CD33 and HLA-DR expression. Also LIN^-^CD33^+^HLA-DR^-^ imMC MDSCs were assessed for expression of neutrophil and basophil markers, CD15 and CD66b.
(TIF)

S2 Fig Gating strategy for T-cell 2 protocol. Labels (in brackets) on top of plots indicate the input gate. (TIF)

S3 Fig Gating strategy for selected T cell phenotypes with the T-cell 1 protocol. (TIF)

S4 Fig Gating strategy for selected B cell phenotypes with the B cell protocol. (TIF)

S5 Fig Gating strategy for additional granulocyte phenotypes with the Granulocyte protocol. (TIF)

S1 Table List of antibodies used in the study. (TIF)

S2 Table Minimal information about a flow cytometry experiment (MIFlowCyt) annotation. (PDF)

S3 Table Cell counts for phenotypes measured from 79 healthy volunteers using the TBNK/M/G protocol. (TIF)

S4 Table Selected list of phenotypes from histograms outlined in Fig. 4. √ = phenotype can be measured from parent population and as cell counts (calculated from data obtained from TBNK/M/G protocol).
(TIF)

S1 Video TBNK phenotypes displayed on radar plots with the rotation of axes. (AVI)

S2 Video Myeloid phenotypes displayed on radar plots with the rotation of axes. (AVI)
