# Supplementary figures and images for: A Method for Identification and Analysis of Non-Overlapping Myeloid Immunophenotypes in Humans
Source: PLoS One. 2015 Mar 23;10(3):e0121546. doi: 10.1371/journal.pone.0121546 (PMC4370675; doi:10.1371/journal.pone.0121546)

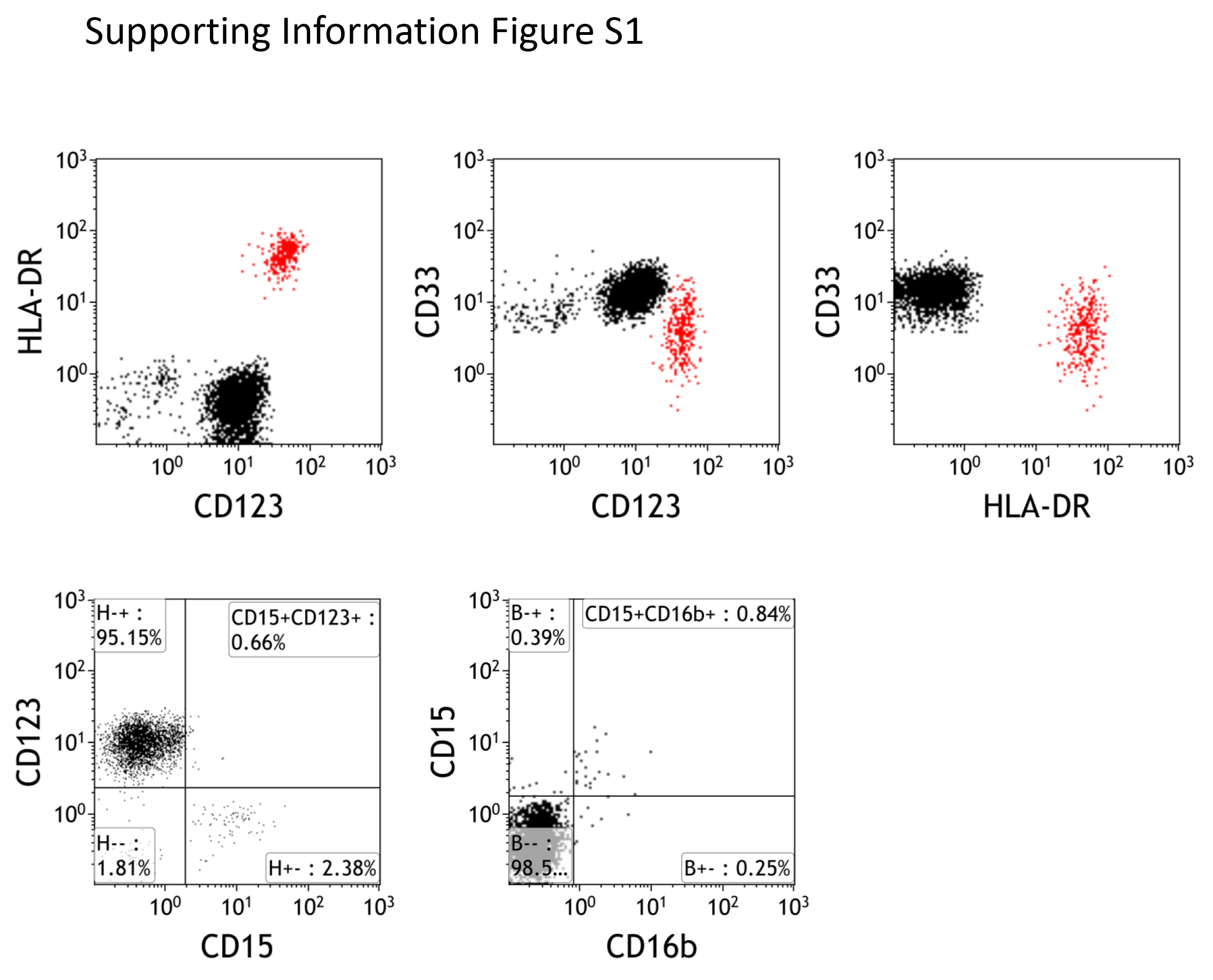

Supplement: S1 Fig — (black) were displayed on the same dot plots as LIN-CD123+CD11c+HLA-DR+ imMC plasmacytoid dendritic cells (red) for comparison of CD33 and HLA-DR expression. Also LIN-CD33+HLA-DR- imMC MDSCs were assessed for expression of neutrophil and basophil markers, CD15 and CD66b. (TIF) [file pone.0121546.s001.tif]

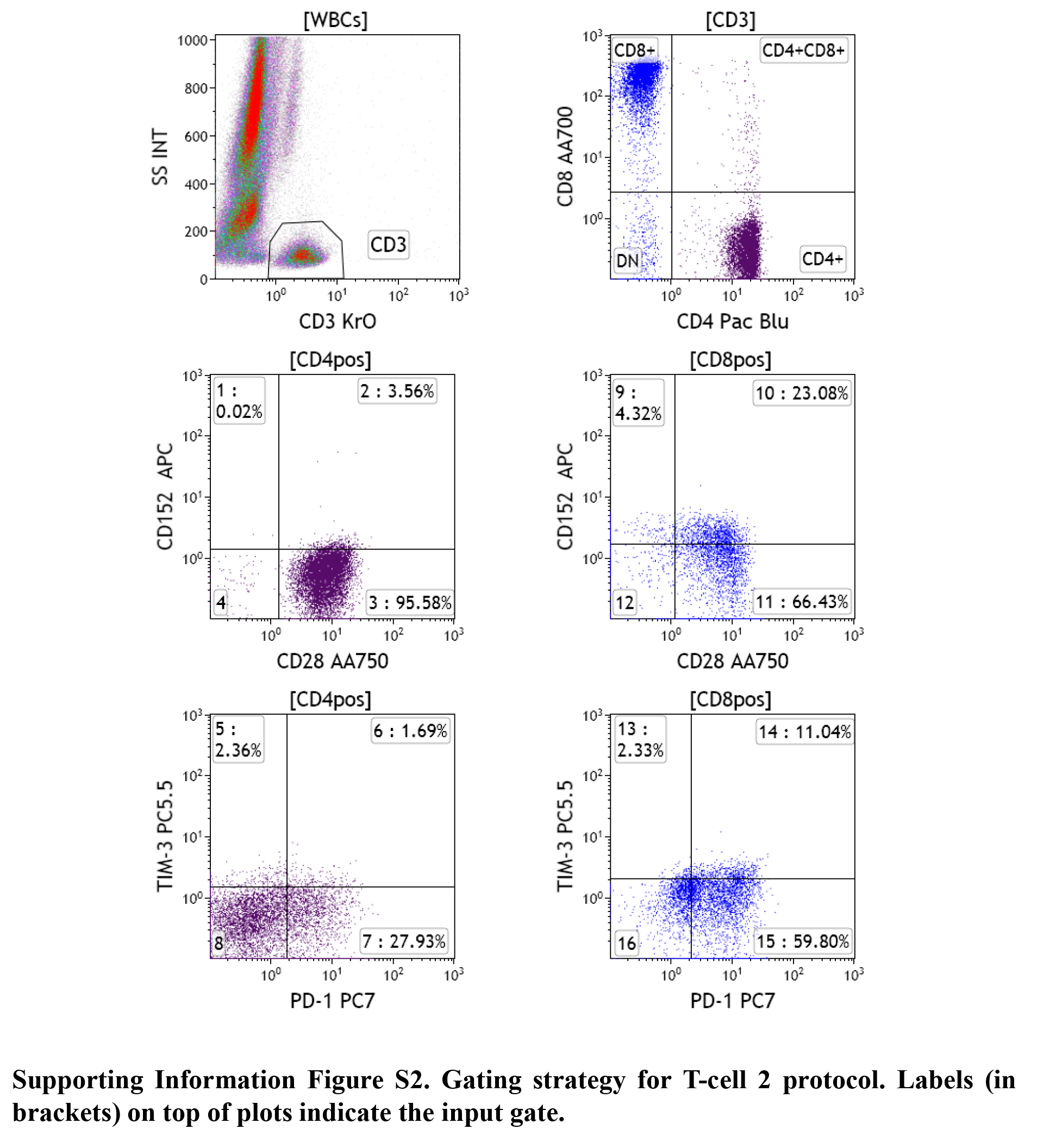

Supplement: S2 Fig — (TIF) [file pone.0121546.s002.tif]

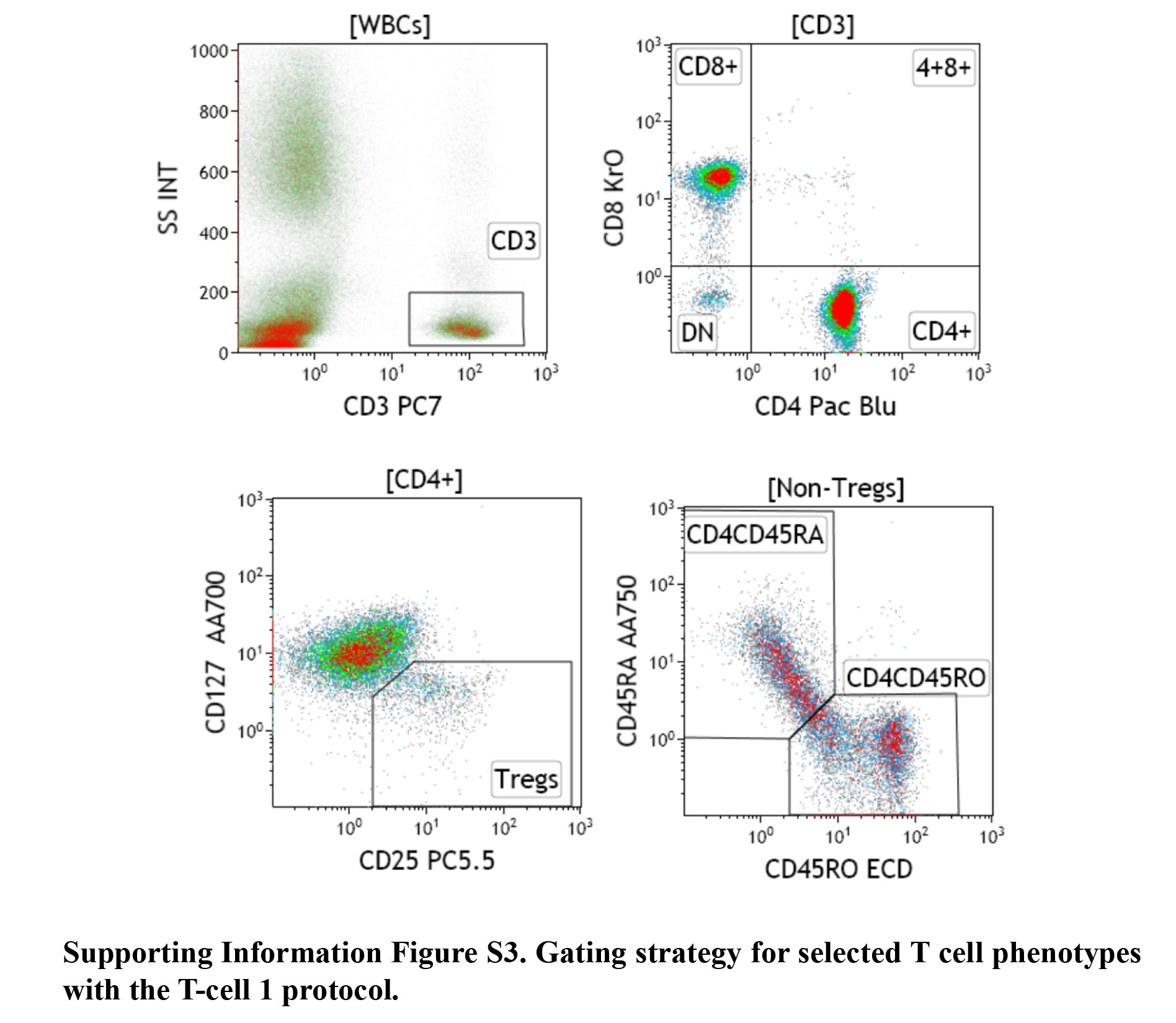

Supplement: S3 Fig — (TIF) [file pone.0121546.s003.tif]

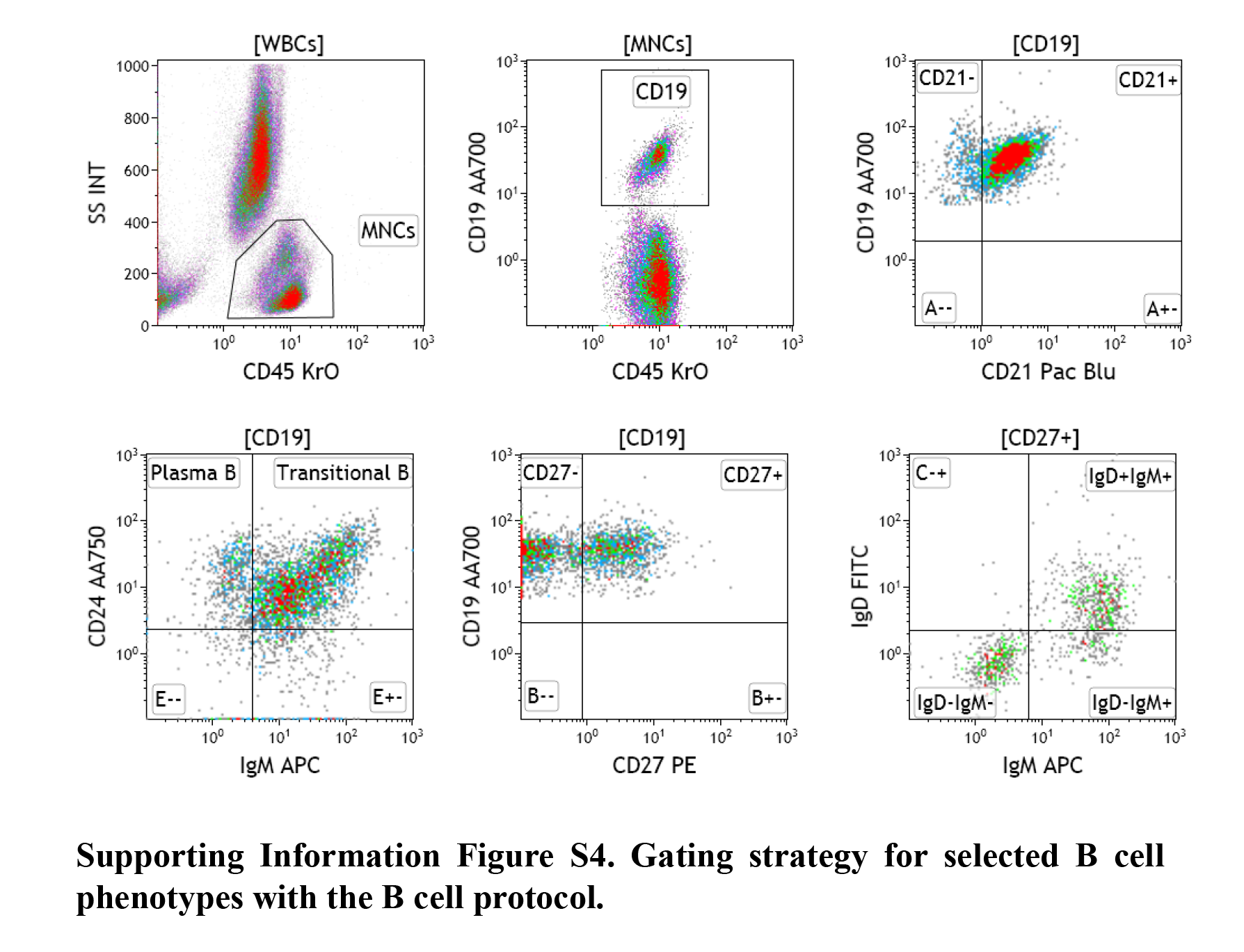

Supplement: S4 Fig — (TIF) [file pone.0121546.s004.tif]

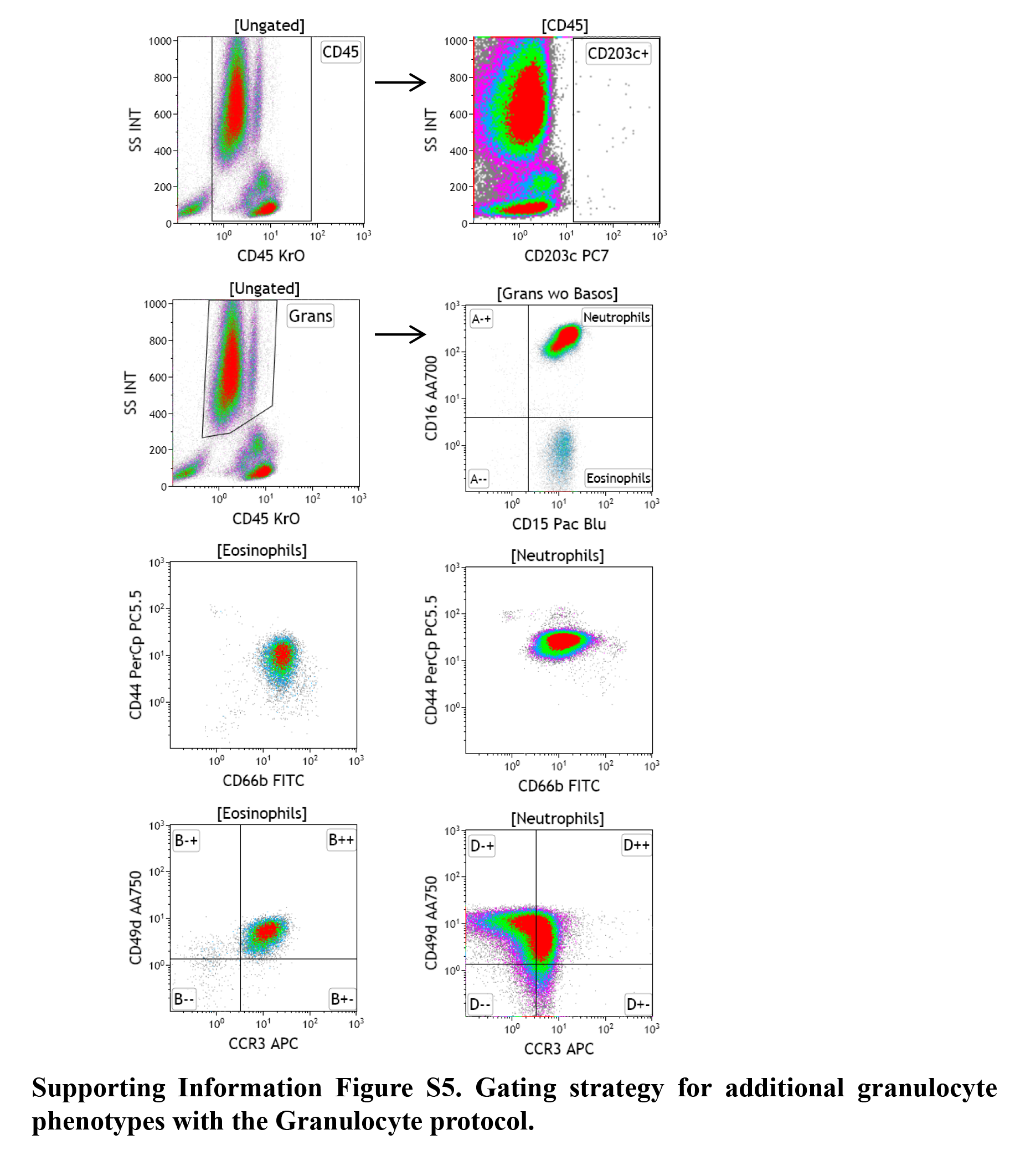

Supplement: S5 Fig — (TIF) [file pone.0121546.s005.tif]

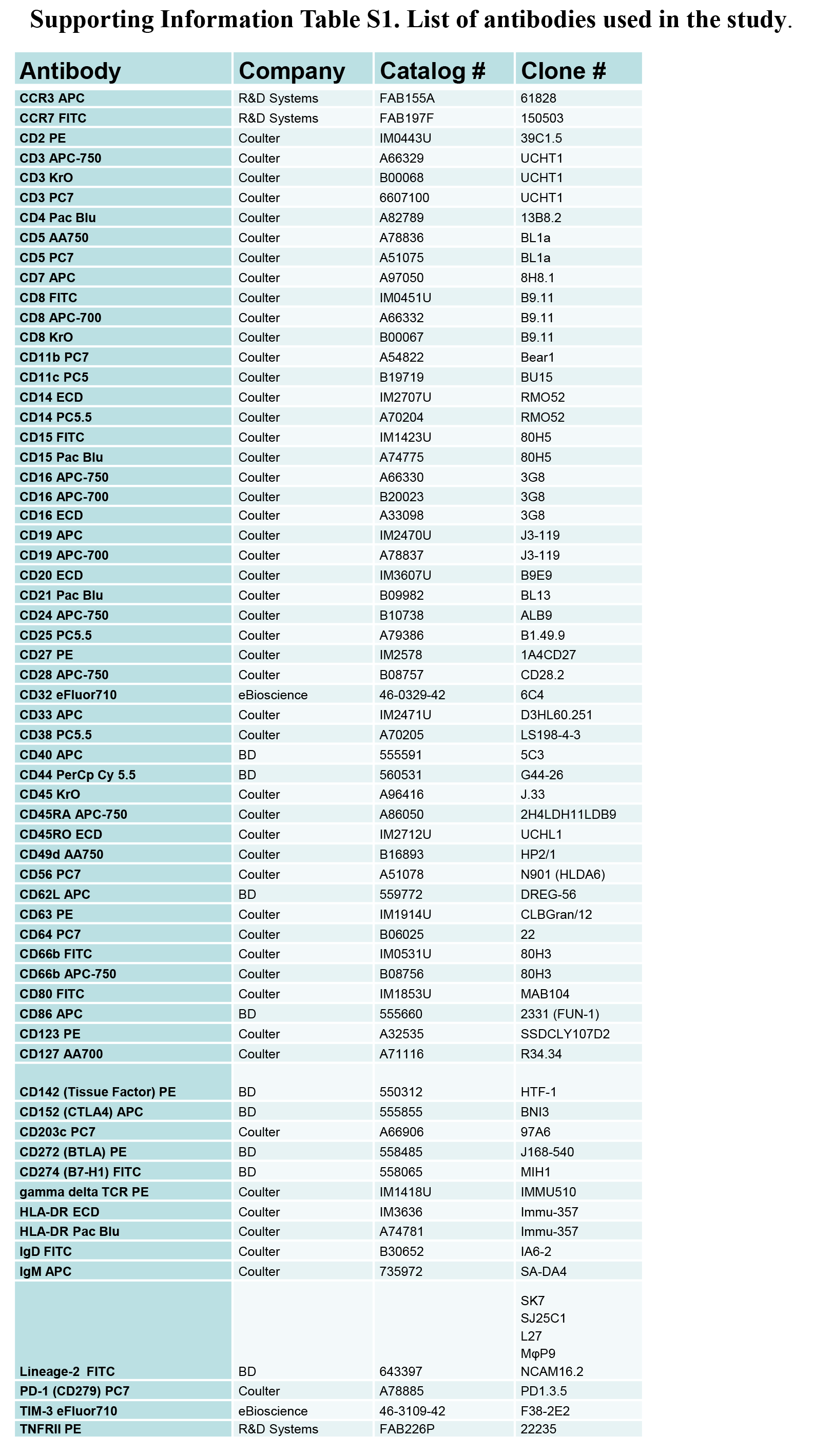

Supplement: S1 Table — (TIF) [file pone.0121546.s006.tif]

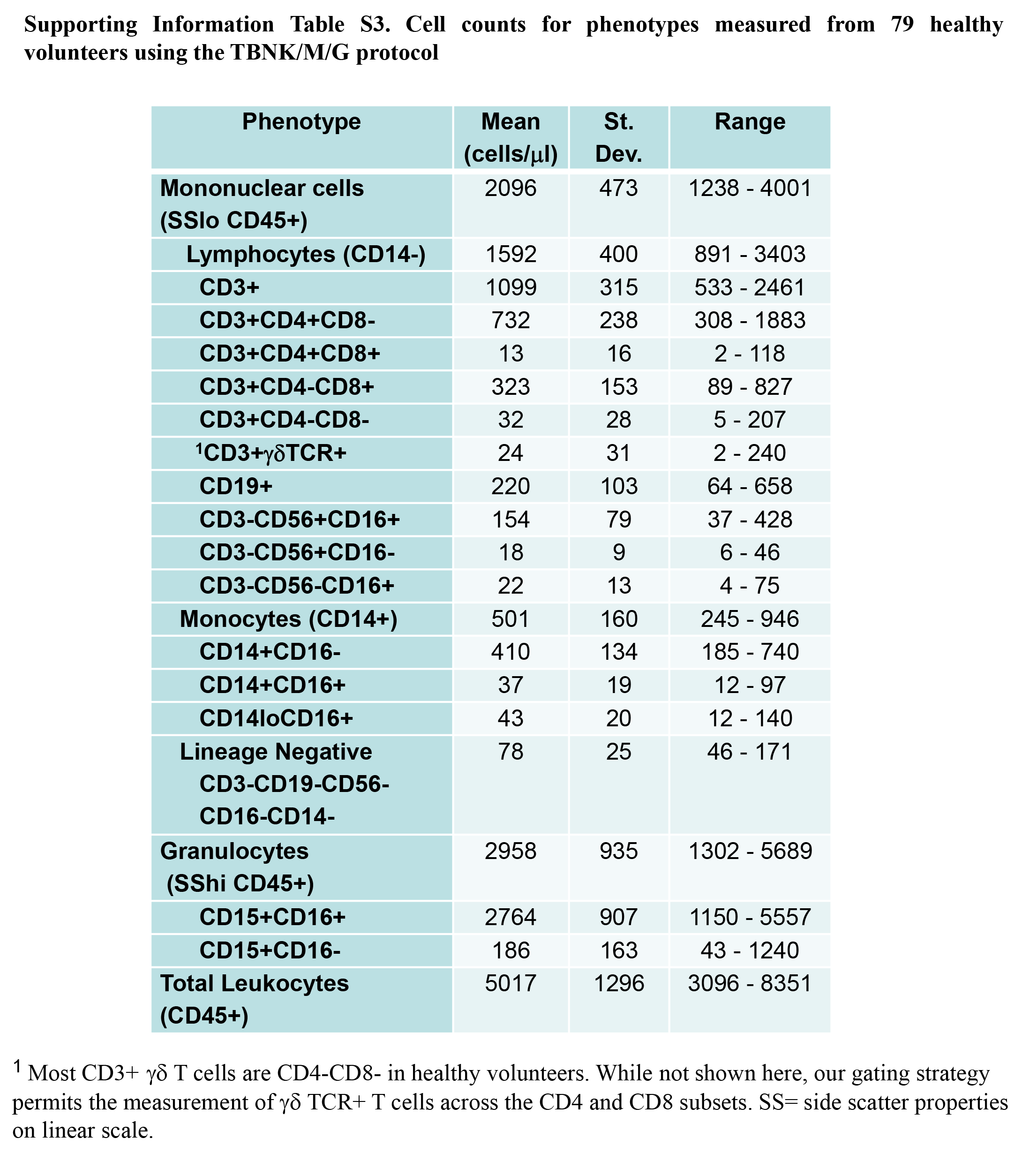

Supplement: S3 Table — (TIF) [file pone.0121546.s008.tif]

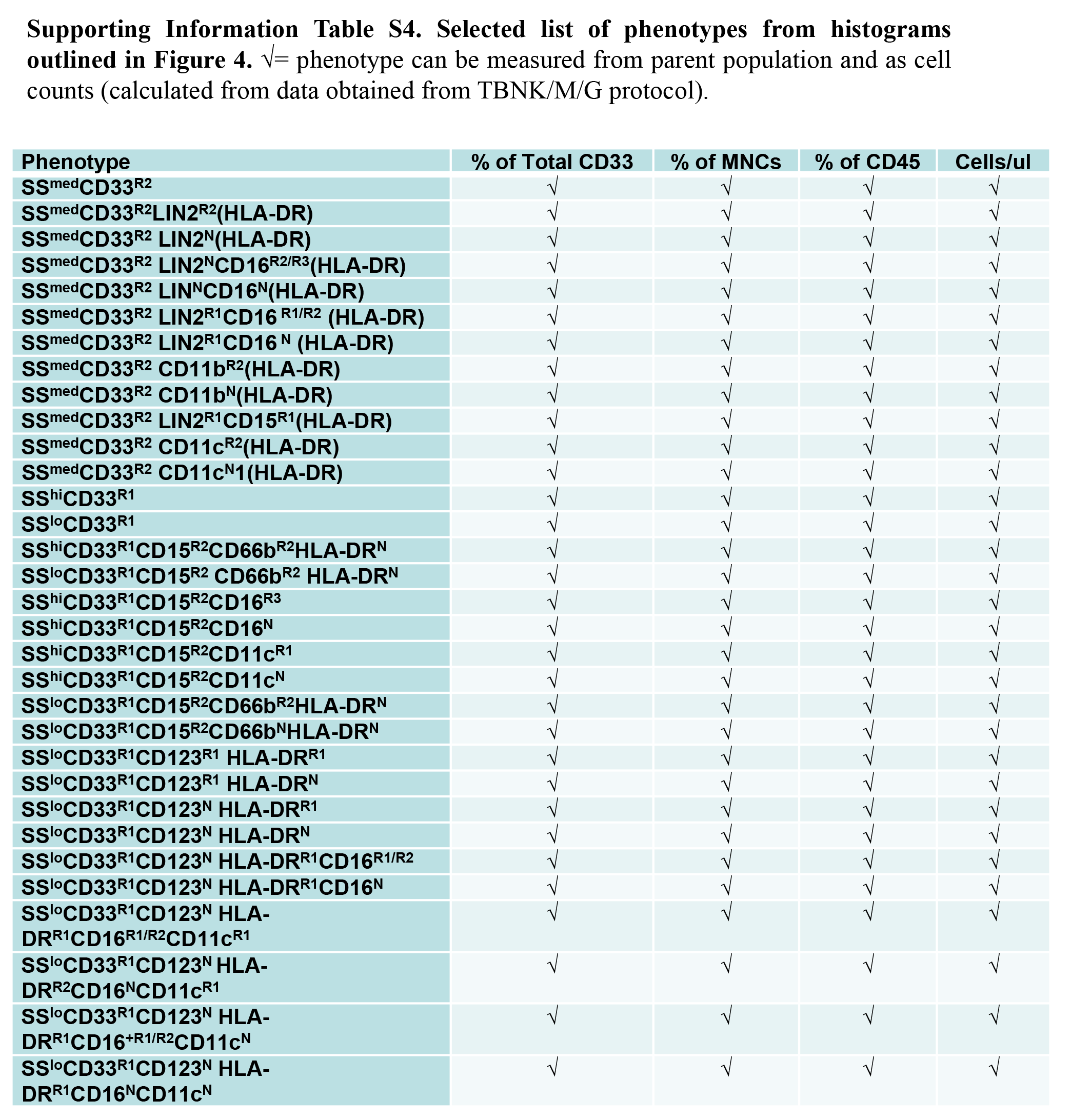

Supplement: S4 Table — √ = phenotype can be measured from parent population and as cell counts (calculated from data obtained from TBNK/M/G protocol). (TIF) [file pone.0121546.s009.tif]
