# Supplementary material for: A Method for Identification and Analysis of Non-Overlapping Myeloid Immunophenotypes in Humans
Source: PLoS One. 2015 Mar 23;10(3):e0121546. doi: 10.1371/journal.pone.0121546 (PMC4370675; doi:10.1371/journal.pone.0121546)
Supplement: S2 Table — (PDF) [file pone.0121546.s007.pdf]

Minimum Information about a Flow Cytometry Experiment (MIFlowCyt) Annotation

1. Experiment Overview
   1.1 Purpose
   The purpose of these sets of experiments is to develop a methodology of identifying and quantifying all of the major leukocytes in human peripheral blood by non-overlapping phenotypes. Our methodology is an attempt to improve the standardization of measuring and assessing human immunophenotypes which is essential for inter-laboratory investigations of translational and clinical research.
   1.2 Keywords
   Myeloid, lymphocyte, leukocyte, human, blood, myeloid derived suppressor, biomarker
   1.3 Experiment Variables
   Blood samples from healthy volunteers were used to establish the protocols. Cancer patient blood and matching bone marrow or pleural effusion samples were collected at various time points before and after treatment.
   1.4 Organization
   1.4.1. Name: Mayo Clinic
   1.4.2. Address: Hilton 2-74, 200 1st St SW, Rochester, MN, 55905
   1.5 Primary Contact
   1.5.1. Name: Allan B. Dietz, Ph.D.
   1.5.2. Email: dietz.allan@mayo.edu
   1.6 Date
   Samples were collected between 12/2013- 6/2014
   1.7 Conclusions
   We have developed an 8-protocol tube multiparameter flow cytometric assay that allows for the assessment of all major leukocyte populations in human blood and other biological samples.
   We have developed a protocol to identify and quantify non-overlapping myeloid phenotypes including myeloid derived suppressor cells.
   1.8 Quality Control Measures
   Verify tubes were used to track instrument settings over time. In cases where the antigens are expressed at low levels or do not have clearly defined positive populations, the position of the positive/negative gate was placed based on either different cell populations within the tube that were clearly negative, or the use of a fluorescence minus one (FMO) control tube.
2. Flow Sample/Specimen Details
   2.1 Sample/Specimen Material Description
   2.1.1 Biological Samples
   2.1.1.1. Biological sample description: 3-10 ml of peripheral blood or bone marrow aspirates collected in K_2EDTA. Pleural effusion

samples were collected in heparinized tubes. Healthy volunteer samples were collected as “waste” samples from the Mayo Clinic Components Laboratory in the division of Transfusion Medicine. Cancer patient samples were collected under Mayo Clinic Human Institutional Review Board approval.

2.1.1.2. Biological sample source description: Homo sapiens

Age: Range: 28-85 years old; Mean: 56.7

Gender: Male=56, Female=23 (healthy volunteers)

2.2 Sample Characteristics

Expected/analyzed types of cells: Leukocytes are distinguished from red blood cells by CD45positivity.

2.3 Sample Treatment Descriptions

100 µl of blood, bone marrow, or pleural fluid were added to each tube and blocked for non-specific antibody binding with 50 µl mouse serum (Sigma-Aldrich, St. Louis, MO) at room temperature for 5 minutes. Prior to the addition of the mouse serum, the pleural fluid was concentrated to 1/10 original volume in phosphate buffered saline (PBS) and for the B cell tube, 3 ml of PBS was added, the tube centrifuged, and the supernatant aspirated. Appropriate antibodies were added to each tube and incubated in the dark for 15 minutes at room temperature. Red blood cells were lysed with the addition of Versa-Lyse (Beckman Coulter, Indianapolis, IN) for at least 20 minutes at room temperature. For the lyse/no wash assay, 100 µl of Flow-Count Flourospheres (Beckman Coulter) were added, mixed and analyzed. For the lyse/wash tubes, samples were centrifuged, washed in PBS containing 1% albumin and 5mM EDTA, and fixed in 1% paraformaldehyde.

2.4 Fluorescence Reagent Description

The list of antibodies can be found in Supplemental Table 3 of the manuscript.

3. Instrument Details

3.1 Instrument Manufacturer

Beckman Coulter

https://www.beckmancoulter.com/wsrportal/wsr/research-and-discovery/index.htm

3.2 Instrument Model

Beckman Coulter Gallios™ Flow Cytometer

http://www.beckmancoulter.com/wsrportal/wsr/research-and-discovery/products-and-services/flow-cytometry/flow-cytometers/gallios/index.htm#2/10//0/25/1/0/asc/2/A94291///0/1//0/

Serial Number AU10414

Technical specification at:

http://www.beckmancoulter.com/wsrportal/wsr/research-and-discovery/products-and-

services/flow-cytometry/flow-cytometers/gallios/index.htm#2/10//0/25/1/0/asc/2/A94291///0/1//0/

3.3 Instrument Configuration and Settings

3.3.1 Flow Cell and Fluidics

The instrument has not been altered.

3.3.2. Light Sources

10 color, 3 laser (488nm Blue, 638nm Red & 405nm Violet) (5 + 3 + 2)

3.3.3. Excitation Optics Configuration

The instrument has not been altered.

3.3.4. Optical filters.

The instrument has not been altered and all filters are original and came with the instrument (April 2012).

3.3.5. Optical detectors

The detector voltages for each protocol (Cytometer Settings Report) are listed on the following pages:

Cytometer Settings Report

Protocol: LNW Final Verify.PRC
Protocol Date: 19-Oct-2012, 14:10:13
Instrument Serial Number: AU10414

	FS	SS	FL1	FL2	FL3	FL4	FL5	FL6	FL7	FL8	FL9	FL10
**Voltage**	367	200	394	404	418	527	461	411	413	480	375	360
**Gain**	5.0	7.5	1.0	1.0	1.0	1.0	1.0	1.0	1.0	1.0	1.0	1.0

	FS	SS	FL1	FL2	FL3	FL4	FL5	FL6	FL7	FL8	FL9	FL10
**Discriminator**	80	OFF	OFF	OFF	OFF	OFF	OFF	OFF	OFF	OFF	OFF	OFF

	FL1	FL2	FL3	FL4	FL5	FL6	FL7	FL8	FL9	FL10
**FL1**		0.7	0.2	0.2	0.3	0	0	0	0	0.4
**FL2**	17.7		5.9	3.5	6.8	0	0	0	0	0
**FL3**	8.3	65.9		2.6	2.9	0	0	0	0	0
**FL4**	0.4	5.4	11.7		0.4	0.3	0.5	0	0	0
**FL5**	0	0.3	0.6	16.8		0	1.3	3.6	0	0
**FL6**	0	0.2	0	0.6	0.4		5.2	4.6	0	0
**FL7**	0	1.4	0	13.8	0.3	31.9		5.2	0	0
**FL8**	0	0.3	0	5.3	11.7	14.8	40.1		0	0
**FL9**	0	0	0	0	0	0	0	0		2.5
**FL10**	2.8	5.7	0	0	0	0	0	0	7.9	

**Lasers**	**Status**	**Shutter**	**Power**	**Current**
**Blue**	On	Closed	21.9 mW	0.42 A
**Red**	On	Closed	25 mW	0.06 A
**Violet**	On	Closed	40.1 mW	0.06 A

Cytometer Settings Report

Protocol: TBNK + Monos + Grans.PRC
Protocol Date: 13-Jan-2014, 18:00:11
Instrument Serial Number: AU10414

	FS	SS	FL1	FL2	FL3	FL4	FL5	FL6	FL7	FL8	FL9	FL10
**Voltage**	207	377	381	376	382	497	476	401	403	470	375	360
**Gain**	7.5	5.0	1.0	1.0	1.0	1.0	1.0	1.0	1.0	1.0	1.0	1.0

	FS	SS	FL1	FL2	FL3	FL4	FL5	FL6	FL7	FL8	FL9	FL10
**Discriminator**	80	OFF	OFF	OFF	OFF	OFF	OFF	OFF	OFF	OFF	OFF	OFF

	FL1	FL2	FL3	FL4	FL5	FL6	FL7	FL8	FL9	FL10
**FL1**		0.7	0.9	0.7	0	0	0	0	0	0.4
**FL2**	12.1		5	2.3	1.8	0	0	0	0	0
**FL3**	7.1	46.9		2.2	2.9	0	0	0	0	0
**FL4**	1	2.8	14.6		1.3	0.3	0.5	0	0	0
**FL5**	0	0.8	3	31.8		0	0.6	1.1	0	0
**FL6**	0	0.2	0	0.1	0.4		5.3	3.9	0	0
**FL7**	0	1.4	0	19.9	0.5	40		4.4	0	0
**FL8**	0	0.3	0	10.7	11.7	11.7	29		0	0
**FL9**	0	0	0	0	0	0	0	0		2.5
**FL10**	2.8	5.7	0	0	0	0	0	0	7.9	

**Lasers**	**Status**	**Shutter**	**Power**	**Current**
**Blue**	On	Closed	21.9 mW	0.42 A
**Red**	On	Closed	25 mW	0.06 A
**Violet**	On	Closed	40.1 mW	0.06 A

Cytometer Settings Report

Protocol: LW Final Verify.PRC
Protocol Date: 04-Jan-2013, 15:45:37
Instrument Serial Number: AU10414

	FS	SS	FL1	FL2	FL3	FL4	FL5	FL6	FL7	FL8	FL9	FL10
**Voltage**	218	279	425	411	409	610	602	557	517	580	361	330
**Gain**	5.0	7.5	1.0	1.0	1.0	1.0	1.0	1.0	1.0	1.0	1.0	1.0
	**FS**	**SS**	**FL1**	**FL2**	**FL3**	**FL4**	**FL5**	**FL6**	**FL7**	**FL8**	**FL9**	**FL10**
**Discriminator**	75	OFF	OFF	OFF	OFF	OFF	OFF	OFF	OFF	OFF	OFF	OFF
	**FL1**	**FL2**	**FL3**	**FL4**	**FL5**	**FL6**	**FL7**	**FL8**	**FL9**	**FL10**		
**FL1**		1.4	0.5	0.1	0.1	0	0	0	0	1.4		
**FL2**	11.9		8.1	1.5	0.7	0	0	0	0	0		
**FL3**	4.9	44.5		0.7	0.3	0	0	0	0	0		
**FL4**	1	9.5	39.8		0.2	0.6	0.5	0	0	0		
**FL5**	0	1	11.3	40.2		0.5	0	3.6	0	0		
**FL6**	0	0	0.4	2	1.6		7.8	13.8	0	0		
**FL7**	0	0	0.4	22.5	0.4	27.7		9.9	0	0		
**FL8**	0	0	0	7	6.3	6	21.1		0	0		
**FL9**	0	0	0	0	0	0	0	0		2.3		
**FL10**	1	1.5	0	0	0	0	0	0	5.9			
**Lasers**	**Status**	**Shutter**	**Power**	**Current**								
**Blue**	On	Closed	21.9 mW	0.42 A								
**Red**	On	Closed	25 mW	0.06 A								
**Violet**	On	Closed	40.1 mW	0.06 A								

Cytometer Settings Report

Protocol: CD4 CD8 Naive&Mem.PRO
Protocol Date: 23-Apr-2013, 13:24:16
Instrument Serial Number: AU10414

	FS	SS	FL1	FL2	FL3	FL4	FL5	FL6	FL7	FL8	FL9	FL10
**Voltage**	218	279	425	431	409	610	602	557	517	580	401	350
**Gain**	5.0	7.5	1.0	1.0	1.0	1.0	1.0	1.0	1.0	1.0	1.0	1.0

	FS	SS	FL1	FL2	FL3	FL4	FL5	FL6	FL7	FL8	FL9	FL10
**Discriminator**	65	OFF	OFF	OFF	OFF	OFF	OFF	OFF	OFF	OFF	OFF	OFF

	FL1	FL2	FL3	FL4	FL5	FL6	FL7	FL8	FL9	FL10
**FL1**		1.4	0.5	0.2	0.1	0	0	0	0	1.4
**FL2**	18.4		8	0.4	0.7	0	0	0	0	0
**FL3**	0	34.5		2.8	0.6	0	0	0	0	0
**FL4**	1	9.5	39.8		0.2	0.6	0.5	0	0	0
**FL5**	0	1	11.3	40.2		0.5	0	1.6	0	0
**FL6**	0	0	0.4	4.3	0.6		9.8	9.5	0	0
**FL7**	0	0	0.4	29.3	0.4	25.7		8.2	0	0
**FL8**	0	0	0	7	6.3	5	21.1		0	0
**FL9**	0	0	0	0	0	0	0	0		2.3
**FL10**	1	1.5	0	0	0	0	0	0	5.9	

**Lasers**	**Status**	**Shutter**	**Power**	**Current**
**Blue**	On	Closed	21.9 mW	0.42 A
**Red**	On	Closed	25 mW	0.06 A
**Violet**	On	Closed	40.1 mW	0.06 A

Cytometer Settings Report

Protocol: T Cell Signaling.PRO
Protocol Date: 28-May-2013, 14:55:44
Instrument Serial Number: AU10414

	FS	SS	FL1	FL2	FL3	FL4	FL5	FL6	FL7	FL8	FL9	FL10
**Voltage**	218	279	425	431	429	650	602	563	517	580	401	350
**Gain**	5.0	7.5	1.0	1.0	1.0	1.0	1.0	1.0	1.0	1.0	1.0	1.0

	FS	SS	FL1	FL2	FL3	FL4	FL5	FL6	FL7	FL8	FL9	FL10
**Discriminator**	75	OFF	OFF	OFF	OFF	OFF	OFF	OFF	OFF	OFF	OFF	OFF

	FL1	FL2	FL3	FL4	FL5	FL6	FL7	FL8	FL9	FL10
**FL1**		1.4	0.2	0.2	0.1	0	0	0	0	1.4
**FL2**	14.9		7.2	1.5	0.7	0	0	0	0	0
**FL3**	4.9	44.5		0.7	0.6	0	0	0	0	0
**FL4**	1	9.5	43.8		0	0.6	0.5	0	0	0
**FL5**	0	1	6.5	14.7		0.5	0	3.6	0	0
**FL6**	0	0	0.4	2	1.6		8.1	13.8	0	0
**FL7**	0	0	0.4	9	0.4	27.7		9.9	0	0
**FL8**	0	0	0	3	6.3	6	27.3		0	0
**FL9**	0	0	0	0	0	0	0	0		2.3
**FL10**	1	1.5	0	0	0	0	0	0	5.9	

**Lasers**	**Status**	**Shutter**	**Power**	**Current**
**Blue**	On	Closed	21.9 mW	0.42 A
**Red**	On	Closed	25 mW	0.06 A
**Violet**	On	Closed	40.1 mW	0.06 A

Cytometer Settings Report

Protocol: B cell.PRC
Protocol Date: 13-Jan-2014, 18:45:19
Instrument Serial Number: AU10414

	FS	SS	FL1	FL2	FL3	FL4	FL5	FL6	FL7	FL8	FL9	FL10
**Voltage**	218	279	415	411	418	610	592	557	517	580	381	330
**Gain**	5.0	7.5	1.0	1.0	1.0	1.0	1.0	1.0	1.0	1.0	1.0	1.0

	FS	SS	FL1	FL2	FL3	FL4	FL5	FL6	FL7	FL8	FL9	FL10
**Discriminator**	75	OFF	OFF	OFF	OFF	OFF	OFF	OFF	OFF	OFF	OFF	OFF

	FL1	FL2	FL3	FL4	FL5	FL6	FL7	FL8	FL9	FL10
**FL1**		1.4	0	0	0.1	0	0	0	0	1.4
**FL2**	12		6.5	1	0.8	0	0	0	0	0
**FL3**	6.2	56.7		1	0.6	0	0	0	0	0
**FL4**	1	9.5	39.8		0.2	0.6	0.5	0	0	0
**FL5**	0	1	11.3	33.5		0	0	1.6	0	0
**FL6**	0	0	0.4	2.3	0		6.9	13.8	0	0
**FL7**	0	0	0.4	27.2	0.4	27.7		9.9	0	0
**FL8**	0	0	0	10.6	10	6	23.1		0	0
**FL9**	0	0	0	0	0	0	0	0		2.3
**FL10**	1	1.5	0	0	0	0	0	0	5.9	

**Lasers**	**Status**	**Shutter**	**Power**	**Current**
**Blue**	On	Closed	21.9 mW	0.42 A
**Red**	On	Closed	25 mW	0.06 A
**Violet**	On	Closed	40.1 mW	0.06 A

Cytometer Settings Report

Protocol: Monocytes-Classical+non.PRC
Protocol Date: 09-Jul-2013, 13:29:57
Instrument Serial Number: AU10414

	FS	SS	FL1	FL2	FL3	FL4	FL5	FL6	FL7	FL8	FL9	FL10
**Voltage**	218	279	425	438	437	710	622	627	517	580	371	330
**Gain**	5.0	7.5	1.0	1.0	1.0	1.0	1.0	1.0	1.0	1.0	1.0	1.0

	FS	SS	FL1	FL2	FL3	FL4	FL5	FL6	FL7	FL8	FL9	FL10
**Discriminator**	75	OFF	OFF	OFF	OFF	OFF	OFF	OFF	OFF	OFF	OFF	OFF

	FL1	FL2	FL3	FL4	FL5	FL6	FL7	FL8	FL9	FL10
**FL1**		1.4	0	0	0.1	0	0	0	0	1.4
**FL2**	14		6.9	0	0.7	0	0	0	0	0
**FL3**	6.2	44.5		0.4	0.6	0	0	0	0	0
**FL4**	1	9.5	62.9		0.2	0.6	0.5	0	0	0
**FL5**	0	1	11.3	21.2		1.4	0	1.6	0	0
**FL6**	0	0	0.4	2.3	1.6		6.9	23.3	0	0
**FL7**	0	0	0	4.6	0.4	14.7		9	0	0
**FL8**	0	0	1	4.6	5.8	2	19.1		0	0
**FL9**	0	0	0	0	0	0	0	0		2.3
**FL10**	1	1.5	0	0	0	0	0	0	5.9	

**Lasers**	**Status**	**Shutter**	**Power**	**Current**
**Blue**	On	Closed	21.9 mW	0.42 A
**Red**	On	Closed	25 mW	0.06 A
**Violet**	On	Closed	40.1 mW	0.06 A

Cytometer Settings Report

Protocol: Granulocytes.PRO
Protocol Date: 23-Apr-2013, 13:36:16
Instrument Serial Number: AU10414

	FS	SS	FL1	FL2	FL3	FL4	FL5	FL6	FL7	FL8	FL9	FL10
**Voltage**	707	219	435	411	409	610	602	557	487	580	391	330
**Gain**	2.0	7.5	1.0	1.0	1.0	1.0	1.0	1.0	1.0	1.0	1.0	1.0

	FS	SS	FL1	FL2	FL3	FL4	FL5	FL6	FL7	FL8	FL9	FL10
**Discriminator**	75	OFF	OFF	OFF	OFF	OFF	OFF	OFF	OFF	OFF	OFF	OFF

	FL1	FL2	FL3	FL4	FL5	FL6	FL7	FL8	FL9	FL10
**FL1**		1.4	0.3	0.1	0.1	0	0	0	0	2.4
**FL2**	10		8.1	0	0.7	0	0	0	0	0
**FL3**	4.9	44.5		0	0.6	0	0	0	1	0
**FL4**	1	9.5	39.8		0.2	0.6	0.5	0	0	0
**FL5**	0	1	11.3	41.2		0.4	0.7	3.6	0	0
**FL6**	0	0	0.4	2.3	1.6		15.9	13.8	0	0
**FL7**	0	0	0	20.7	0.4	16.7		11.9	0	0
**FL8**	0	0	0	8.6	5.2	6	41.2		0	0
**FL9**	0	0	0	0	0	0	0	0.1		3.3
**FL10**	1	0	0	0	0	0	0	3	5.9	

**Lasers**	**Status**	**Shutter**	**Power**	**Current**
**Blue**	On	Closed	21.9 mW	0.42 A
**Red**	On	Closed	25 mW	0.06 A
**Violet**	On	Closed	40.1 mW	0.06 A

Cytometer Settings Report

Protocol: MDSCs.PRC
Protocol Date: 23-Apr-2013, 13:38:51
Instrument Serial Number: AU10414

	FS	SS	FL1	FL2	FL3	FL4	FL5	FL6	FL7	FL8	FL9	FL10
**Voltage**	208	137	411	430	432	690	654	627	517	610	401	330
**Gain**	5.0	7.5	1.0	1.0	1.0	1.0	1.0	1.0	1.0	1.0	1.0	1.0

	FS	SS	FL1	FL2	FL3	FL4	FL5	FL6	FL7	FL8	FL9	FL10
**Discriminator**	75	OFF	OFF	OFF	OFF	OFF	OFF	OFF	OFF	OFF	OFF	OFF

	FL1	FL2	FL3	FL4	FL5	FL6	FL7	FL8	FL9	FL10
**FL1**		0.8	0	0	0.1	0	0	0	0	1.4
**FL2**	16.8		5.6	0.8	0.7	0	0	0	0	0
**FL3**	7.2	52.1		0.4	0.4	0	0	0	0	0
**FL4**	1	9.5	53.6		0.2	0.4	0.5	0	0	0
**FL5**	0	1	7.3	30.2		0.1	0	3.6	0	0
**FL6**	0	0	0.4	2.3	0		13.4	19.8	0	0
**FL7**	0	0	0	11.1	0	12.3		9.9	0	0
**FL8**	0	0	0	5.9	4.5	2.7	23.1		0	0
**FL9**	0	0	0	0	0.1	0	0	0		2.3
**FL10**	1	0	0	0	0	0	0	0	5.9	

**Lasers**	**Status**	**Shutter**	**Power**	**Current**
**Blue**	On	Closed	21.9 mW	0.42 A
**Red**	On	Closed	25 mW	0.06 A
**Violet**	On	Closed	40.1 mW	0.06 A

Cytometer Settings Report

Protocol: Monocytes-Signaling.PRO
Protocol Date: 24-Jun-2013, 17:29:11
Instrument Serial Number: AU10414

	FS	SS	FL1	FL2	FL3	FL4	FL5	FL6	FL7	FL8	FL9	FL10
**Voltage**	218	279	415	431	439	610	622	597	517	580	371	330
**Gain**	5.0	7.5	1.0	1.0	1.0	1.0	1.0	1.0	1.0	1.0	1.0	1.0

	FS	SS	FL1	FL2	FL3	FL4	FL5	FL6	FL7	FL8	FL9	FL10
**Discriminator**	75	OFF	OFF	OFF	OFF	OFF	OFF	OFF	OFF	OFF	OFF	OFF

	FL1	FL2	FL3	FL4	FL5	FL6	FL7	FL8	FL9	FL10
**FL1**		1.4	0	0	0.1	0	0	0	0	1.4
**FL2**	24.2		0.8	0	1.1	0	0	0	0	0
**FL3**	12.6	58.2		0.4	0.6	0	0	0	0	0
**FL4**	1	1	1.1		0.2	0.6	0.5	0	0	0
**FL5**	0	1	1.1	41.2		1.4	0	1.3	0	0
**FL6**	0	0	0	2.3	0.2		6.9	19.8	0	0
**FL7**	0	0	0	19.5	0.2	15.2		9.9	0	0
**FL8**	0	0	0	8.6	4.2	1.2	19.1		0	0
**FL9**	0	0	0	0	0	0	0	0		2.3
**FL10**	1	0	0	0	0	0	0	0	6.9	

**Lasers**	**Status**	**Shutter**	**Power**	**Current**
**Blue**	On	Closed	21.9 mW	0.42 A
**Red**	On	Closed	25 mW	0.06 A
**Violet**	On	Closed	40.1 mW	0.06 A

3.4 Other Relevant Instrument Details

Kaluza Software Analysis reference: http://www.beckmancoulter.com/wsrportal/wsr/research-and-discovery/products-and-services/flow-cytometry/software/kaluza-analysis-software/index.htm

4. Data Analysis Details

4.1 List-mode Data Files

LMD data files can be obtained by contacting Dr. Allan Dietz after this work has been published.

4.2 Compensation Description

The compensation matrix for each protocol is listed on the Cytometer Settings Report.

4.3 Data Transformation Details

4.3.1 Purpose of Data Transformation

Visualization and gating

4.3.2 Data Transfomation Description

-Visualization of dot plots in Kaluza software analysis of LMD files were displayed by density or by gate color.

- The coordinates of the radar plots are listed in each LMD analysis file.

4.4 Gating (Data Filtering) Details

The gating strategies for each of the protocols are outlined in the manuscript.
